# Evidence for a Modulatory Effect of a 12‐Week Pomegranate Juice Intervention on the Transcriptional Response in Inflammatory Bowel Disease Patients Reducing Fecal Calprotectin Levels: Findings From a Proof‐of‐Principle Study

**DOI:** 10.1002/mnfr.70067

**Published:** 2025-04-21

**Authors:** Ilaria Minato, Pedro Mena, Luigi Ricciardiello, Eleonora Scaioli, Andrea Belluzzi, Enrica Rotondo, Eleonora Derlindati, Barbara Montanini, Costanza Michelini, Nicole Tosi, Vicente Agullò Garcià, Gianfranco Picone, Carlo Mengucci, Sara Dobani, Paloma Salamanca, Alice Rosi, Margherita Dall'Asta, Letizia Bresciani, Claudio Curti, Enzo Spisni, Alessandra Dei Cas, Alessandra Bordoni, Francisco A. Tomás‐Barberán, Lynnette R. Ferguson, Daniele Del Rio, Francesca Danesi

**Affiliations:** ^1^ Department of Chemistry Life Sciences and Environmental Sustainability University of Parma Parma Italy; ^2^ Interdepartmental Center for Innovation in Health Products Biopharmanet‐TEC University of Parma Parma Italy; ^3^ Department of Food and Drug University of Parma Parma Italy; ^4^ Microbiome Research Hub University of Parma Parma Italy; ^5^ IRCCS ‐ St. Orsola‐Malpighi Hospital Bologna Italy; ^6^ Department of Medical and Surgical Sciences University of Bologna Bologna Italy; ^7^ Department of Agricultural and Food Sciences University of Bologna Cesena Italy; ^8^ Department of Animal Science, Food and Nutrition Università Cattolica del Sacro Cuore Piacenza Italy; ^9^ Department of Biological, Geological, and Environmental Sciences University of Bologna Bologna Italy; ^10^ Department of Medicine and Surgery ‐ Division of Endocrinology and Metabolic Diseases University of Parma Parma Italy; ^11^ Quality, Safety, and Bioactivity of Plant Foods CEBAS–CSIC Murcia Spain; ^12^ Department of Nutrition Faculty of Medical and Health Sciences University of Auckland Auckland New Zealand

**Keywords:** ellagitannins, fecal calprotectin, inflammatory bowel disease, pomegranate juice, urolithins

## Abstract

This study aimed at investigating the effects of pomegranate juice (POMJ) consumption on inflammatory biomarkers and gene expression in patients with inflammatory bowel disease (IBD) in clinical remission. In this randomized, placebo‐controlled trial, 16 subjects with IBD in remission consumed POMJ or placebo for 12 weeks. POMJ consumption significantly reduced fecal calprotectin (FC) and plasma endotoxin levels. Transcriptomic analysis of peripheral blood mononuclear cells revealed upregulation of genes involved in mucosal immunity, including aryl hydrocarbon receptor (*AHR*), neutrophil cytosolic factor 4 (*NCF4*), and nuclear factor, interleukin 3 regulated (*NFIL3*). Urolithin metabotypes were predominantly of the B type, associated with intestinal dysbiosis. No significant changes were observed in serum inflammatory markers or colonic mucosal cytokine expression. POMJ consumption reduced markers of intestinal inflammation and modulated gene expression related to mucosal immunity and barrier function in patients with IBD. These findings suggest the potential of POMJ as a beneficial dietary intervention for maintaining remission in IBD, highlighting the promise of targeted nutritional strategies in managing chronic inflammatory conditions. Further research is needed to elucidate the long‐term clinical implications of these molecular changes.

**Trial Registration:**

ClinicalTrials.gov identifier: NCT03000101.

## Graphical abstract

This study investigated how pomegranate juice affects inflammation in patients with inflammatory bowel disease who are currently in remission. After 12 weeks of drinking pomegranate juice, patients showed reduced markers of intestinal inflammation. The juice's beneficial effects appear to work through two mechanisms: locally in the gut, where metabolites of pomegranate compounds improve intestinal barrier function, and systemically, where they help regulate immune responses.

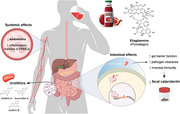

**Abbreviations**
AHR: aryl hydrocarbon receptor
CD: Crohn's disease
CDAI: Crohn's disease activity index
CRP: C‐reactive protein
DEG: differentially expressed gene
ET: ellagitannin
FC: fecal calprotectin
FFQ: food frequency questionnaire
FRAP: ferric reducing antioxidant power
GI: gastrointestinal
IBD: inflammatory bowel disease
IPA: ingenuity pathway analysis
IQR: interquartile range
MDD: minimum detectable dose
MES: Mayo endoscopic subscore
NCF4: neutrophil cytosolic factor 4
NFIL3: nuclear factor interleukin 3 regulated
PBMC: peripheral blood mononuclear cell
PCA: principal component analysis
POMJ: pomegranate juice
qPCR: quantitative polymerase chain reaction
SCCAI: simple clinical colitis activity index
TAC: total antioxidant capacity
TMAO: trimethylamine N‐oxide
TNF‐α: tumor necrosis factor‐ α
UC: ulcerative colitis
UHPLC: ultra‐high performance liquid chromatography

## 1 Introduction

Among chronic inflammatory diseases, the occurrence of inflammatory bowel disease (IBD) has steadily risen over time in the world population and, in particular, in children [1]. This increasing prevalence underscores the urgent need for new strategies to mitigate the burden of IBD.

IBD is an umbrella term used to describe lifelong conditions that cause chronic inflammation of the gastrointestinal (GI) tract. The two most common forms of IBD are Crohn's disease (CD) and ulcerative colitis (UC). Currently, monitoring, treatment, and therapy for CD and UC are similar [2], and the long‐term maintenance of remission is considered a major goal in clinical practice for patients with IBD. Stable pharmacological therapy with immunosuppressive and immune‐modulating agents remains the most effective way to reduce relapse episodes in subjects with IBD [3]. Although evidence for dietary interventions in maintaining IBD remission remains limited [4], consumption of foods rich in (poly)phenols might be effective as complementary support for the maintenance of remission and improving patient quality of life on a long‐term basis. Many studies, mainly conducted in preclinical models, have focused on the therapeutic potential of phytochemical‐rich foods such as pomegranate in chronic inflammatory diseases [5, 6]. However, evidence from randomized clinical trials on their effectiveness in humans remains scarce.

Among the various (poly)phenols found in foods, ellagitannins (ETs), the most abundant class of (poly)phenols in pomegranate and also present in berries, walnuts, and other nuts [7], have shown particular promise in chronic inflammatory conditions [8, 9, 10]. As for other bioactive compounds, ETs are extensively metabolized, both in intestinal tissues and by gut microbiota, before their absorption through the intestinal barrier [11, 12]. This considerable metabolism in the gut microenvironment involves complex enzymatic processes and releases an array of ET metabolites that can act locally in the intestine, shaping the ecology of the gut microbiota, protecting against inflammation, and exerting their effects on other organs through the bloodstream [13, 14]. The main metabolites detected in vivo following the release of ellagic acid from ETs are urolithins (urolithin A, isourolithin A, urolithin B, and their phase II conjugates), which can reach relatively high (micromolar) concentrations in different body fluids and tissues [13, 15]. The reciprocal interactions between ETs and the gut may be of particular interest in modulating inflammation in IBD.

Given the promising evidence from preclinical models of IBD on ETs and their metabolization in the gut, there is a clear need to investigate their potential benefits in humans. To this end, our study addresses a critical gap in IBD management: the need for non‐pharmacological strategies to mitigate rising levels of fecal calprotectin (FC) in patients at high risk of relapse. FC, a neutrophil‐derived protein, serves as a surrogate marker of mucosal inflammation and strongly correlates with endoscopic and histological measures of inflammation [16, 17]. Despite being in stable clinical remission, as determined by clinical activity indices, patients with elevated FC (≥ 100 µg/g) face an increased risk of clinical relapse. We conducted a randomized controlled trial to investigate the effects of a 12‐week intervention with 100% pomegranate juice (POMJ), a naturally rich source of ETs, on FC levels (primary outcome) and other inflammatory markers in plasma and intestinal mucosa (secondary outcomes) in these high‐risk patients. As additional secondary outcomes, we assessed the transcriptional responses of circulating peripheral blood mononuclear cells (PBMCs), which may play a role in maintaining long‐term remission in IBD and are recognized as potential targets for novel therapeutic strategies within immunometabolism [18]. To explore the complex relationship between ET intake and inflammatory status, we also clustered participants based on their urolithin‐phenotype changes in response to the intervention. This comprehensive approach aims to elucidate POMJ's potential as a dietary intervention to modulate subclinical inflammation and potentially prolong remission in patients with IBD.

## 2 Experimental Section

### 2.1 Materials

Pure 100% POMJ (Wonderful cultivar) was sourced from Gat Foods (M.P., Hefer, Israel). The POMJ was chosen from pure juices of three different cultivars (Wonderful, Hicaznar, and Mollar de Elche) and juices from concentrate at different dilutions (50% to 100%) on the basis of sensory quality (appearance, taste, and aroma) by a panel of 20 healthy volunteers prior to the intervention study. Placebo (water, sucrose, and citric acid monohydrate) was designed to be similar in moisture, carbohydrates, total soluble solids, titratable acid, and energy to the selected POMJ. Both experimental beverages were supplied by Conserve Italia (Bologna, Italy) in a 125 mL white juice box with a drinking straw (Tetra Brik Aseptic, Tetra Pak, Lund, Sweden). All chemicals and solvents were obtained from Sigma‐Aldrich Co. (St. Louis, MO, USA) unless otherwise stated.

### 2.2 Chemical Characterization, Profile and Content of (Poly)Phenols in Experimental Beverages

Nutrient contents were determined using official methods [19, 20]. Energy was determined by calculation. The POMJ and placebo (poly)phenols were extracted and analyzed as described in a previous study [21], with minor modifications provided in the Supporting Information. Identification was carried out using MS^2^ and MS^3^ information, as reported in **Table** **S1**.

### 2.3 Participants and Study Overview

This trial was carried out in accordance with the Declaration of Helsinki and approved by the Ethics Committee of St. Orsola‐Malpighi Hospital (Bologna, Italy) on September 13, 2016 (89/2016/U/Sper). This study was registered at clinicaltrials.gov as NCT03000101 (https://clinicaltrials.gov/study/NCT03000101). Screening and study visits were conducted at the Gastroenterological Unit of St. Orsola‐Malpighi Hospital. All the participants provided written informed consent before participating in the study.

The experimental design of this double‐blind, randomized, placebo‐controlled trial has been described in extenso in Scaioli et al. (2019) [22]. Briefly, patients with IBD in stable clinical remission with a high risk of clinical relapse were asked to consume POMJ over a 12‐week period in order to test for systemic and mucosal changes in biochemical and molecular inflammatory response markers relative to a control group receiving a placebo. Eligible participants were asymptomatic subjects aged between 18 and 80 years with UC or CD (the latter mainly involving the sigmoid colon and rectum) in stable clinical remission (Simple Clinical Colitis Activity Index (SCCAI) ≤ 2 for patients with UC; CD Activity Index (CDAI) < 150 for patients with CD) [23, 24, 25] for at least 3 months with FC levels ≥ 100 µg/g. Patients undergoing concomitant stable therapies for UC (mesalamine, immunomodulators, and/or biological drugs) without modifications in the previous 3 months were included. The exclusion criteria were as follows: (i) recent use of steroids (< 2 months) or other experimental drugs (< 3 months); (ii) use of anticoagulants; (iii) probiotic use; (iv) pregnancy or breastfeeding; (v) known or suspected hypersensitivity to pomegranate fruit or juice; and (vi) serious comorbidities.

**FIGURE 1:**
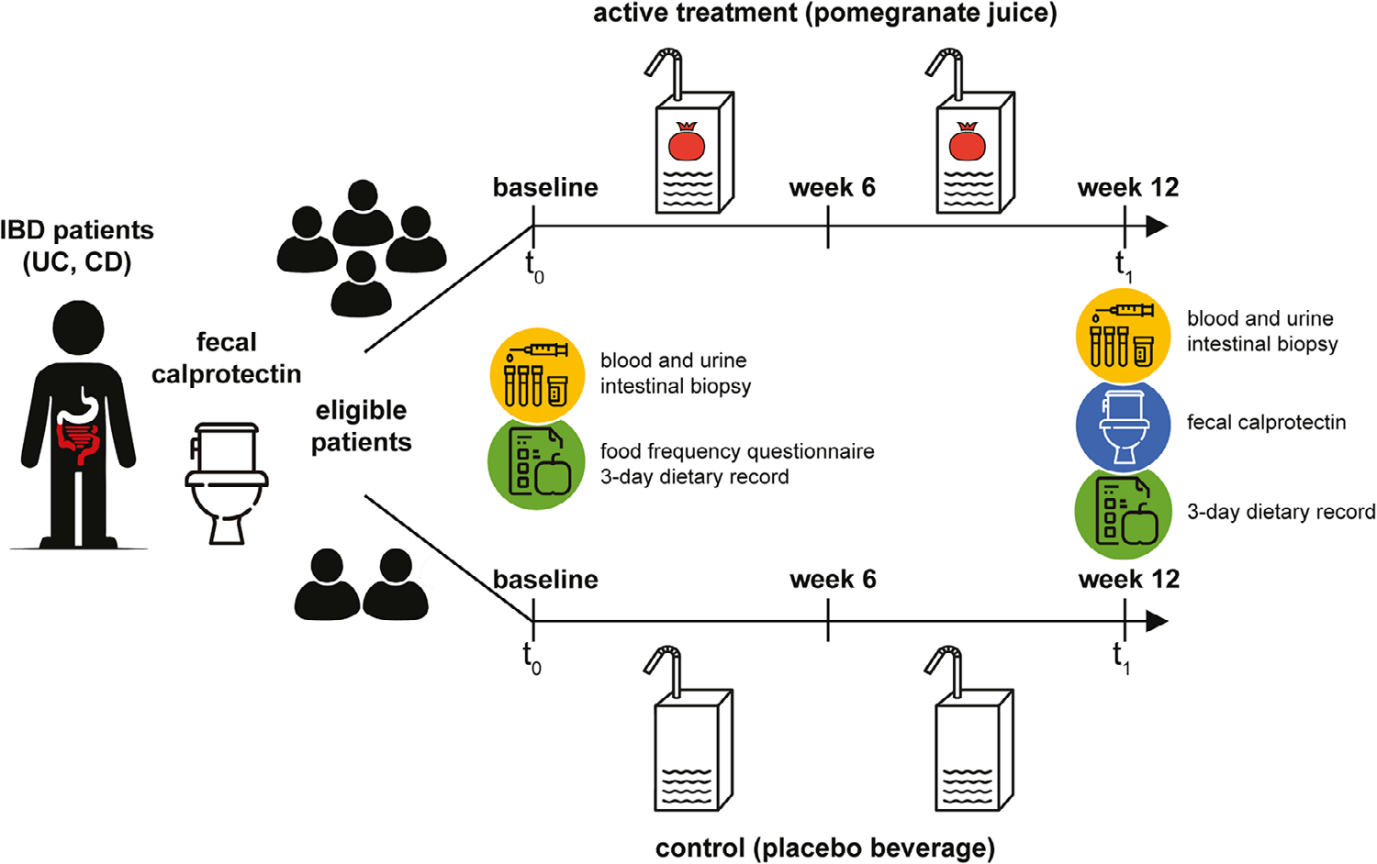
Scheme of the study design. Adapted from Scaioli et al. (2019) [22] licensed under the Creative Commons Attribution 4.0 International License. CD: Crohn's disease; IBD: inflammatory bowel disease; UC: ulcerative colitis.

Patients who satisfied the inclusion criteria underwent laboratory tests and total colonoscopy with multiple biopsies at baseline (t_0_). They were then randomized in a 2:1 ratio using Random Allocation Software (version 1.0.0) [26] with a parallel block design [27] to receive active supplementation (125 mL of POMJ, after a light meal if possible, twice daily) or placebo (125 mL, after a light meal if possible, twice daily) for 12 weeks. The 2:1 randomization scheme was chosen to increase enrollment and safely obtain data on active treatment (POMJ) in patients with IBD in stable clinical remission. Both beverages were packaged indistinguishably and labeled with participant ID numbers to maintain double‐blinding of investigators and participants throughout the trial. Clinical assessments, FC measurements, and fasting blood and urine tests were repeated after intervention (t_1_). In addition, a 6‐week visit was conducted for all participants to record changes according to the exclusion criteria during the entire study period. An overview of the study design is shown in **Figure** **1**.

The primary outcome of the present two‐armed study with parallel groups was the change in FC after the 12‐week intervention. Secondary outcomes included transcriptomic changes in peripheral PBMCs and intestinal biopsies and changes in the levels of circulating inflammatory markers. Pomegranate ET‐derived metabolites in plasma and urine samples were also assessed.

During enrollment, the participants’ dietary habits were assessed using a semi‐quantitative food frequency questionnaire (FFQ). Subjects were instructed by a nutritionist to maintain their dietary habits largely unaltered, except for twice‐daily beverage supplementation, and to follow a diet low in trimethylamine *N*‐oxide (TMAO)‐producing foods, such as fish and shellfish, eggs, red and processed meat, and to avoid the consumption of dairy products, poultry, and whole foods for 24 h before sampling at *t*_0_ and *t*_1_. Participants were also asked to restrict their consumption of ET‐containing foodstuffs and supplements throughout the study to standardize the level of daily ET exposure and minimize the influence of other ET sources during the intervention period. To obtain information regarding dietary intake, participants filled out a 3‐day food diary (3‐DFD) 3 days before each sampling day at the beginning and end of the study. These data were collected to check for omissions or compliance and support the evaluation of ET‐derived metabolites, as plasma and urine metabolite composition reflects dietary habits.

### 2.4 Assessment of Dietary Habits

On the day of allocation, a semi‐quantitative FFQ was administered to participants by trained laboratory personnel to evaluate the dietary total antioxidant capacity (TAC) [28]. The dietary TAC value was calculated as the sum of the ferric reducing‐antioxidant power (FRAP) of each consumed food item and was expressed in terms of mmol of Fe^2+^ equivalents per kg of food.

### 2.5 Sample Collection and Processing

Stool specimens were collected at home from all subjects at enrollment and after 12 weeks of intervention, within 24 h before each endoscopy, using a stool container. The samples were stored at 2°C–8°C until FC measurement.

After overnight fasting, urine samples were collected from the study participants in sterile urine collection cups at baseline and at the end of the intervention. Samples were aliquoted into microcentrifuge tubes and stored at −80°C until analysis for pomegranate ET‐derived metabolites.

To determine changes in circulating inflammatory markers and TMAO levels and to measure pomegranate‐derived ET metabolites, peripheral blood samples were collected via venipuncture from participants at *t*_0_ and *t*_1_ into sterile vacutainers containing EDTA as an anticoagulant (BD Vacutainer K2 EDTA tubes; Becton Dickinson and Company, Franklin Lakes, NJ, USA). Plasma samples were separated from the blood by centrifugation at 1300 × *g* for 10 min in a refrigerated centrifuge without brake. Separated samples were aliquoted and stored at −80°C for subsequent testing (inflammatory markers and ET‐derived metabolites). To investigate transcriptomic changes in response to the 12‐week intervention, PBMCs were purified from whole blood samples using SepMate tubes (STEMCELL Technologies, Vancouver, BC, Canada) with Lymphoprep as a density gradient medium, following the manufacturer's instructions. PBMC pellets were frozen and stored at −80°C until RNA extraction.

Colon mucosal biopsies for transcriptomic evaluations were obtained at *t*_0_ and *t*_1_ from the sigmoideum of the subjects using endoscopy at St. Orsola‐Malpighi Hospital, Bologna, Italy. Two mucosal biopsies were performed, up to 20 cm from the anal verge, at each time point. Biopsy specimens were immediately transferred to 1.5 mL microcentrifuge tubes prefilled with 1.0 mL RNA*later* solution (Thermo Fisher Scientific Inc., San José, CA, USA), stored overnight at 4°C, then RNA preservative solution was removed by careful pipetting, and biopsy material was rapidly transferred to −80°C for storage until RNA extraction.

### 2.6 FC Measurement

Measurements of FC were performed at enrollment and after 12 weeks of intervention using a CalFast immunochromatographic assay (Eurospital, Trieste, Italy) according to the manufacturer's instructions. FC levels were quantified using a CalFast reader (Eurospital) and expressed as µg/g. The assay has a lower detection limit of 15 µg/g. Results were provided by the automated reader in a range of 50 to 300 µg/g; if the reading was below or above this range, the result was reported as < 50 µg/g or > 300 µg/g, respectively.

### 2.7 Clinical and Endoscopic Assessments

The disease‐specific activity was documented by a gastroenterologist at *t*_0_ and *t*_1_ using the SCCAI for patients with UC or the CDAI for patients with CD [23, 24].

The Mayo Endoscopic Subscore (MES) [29] was only assessed in subjects who underwent endoscopy at *t*_0_ and *t*_1_. A cutoff value ≥ 2 was used to discriminate the presence of endoscopic inflammation at baseline (*t_0_*) and post‐intervention (*t*_1_) [25].

### 2.8 Quantification of Levels of Inflammatory Markers in Plasma

#### 2.8.1 C‐Reactive Protein (CRP) Measurement

Plasma C‐reactive protein (CRP) levels were assessed using Quantikine ELISA Human CRP Immunoassay (R&D Systems Inc., Minneapolis, MN, USA) according to the manufacturer's recommendations. The minimum detectable dose (MDD) of CRP in the assay is shown in **Table** **S2**. A standard curve was created (0.78–50 ng/mL), and four‐parameter logistic (4PL) regression fitting was used to estimate the CRP concentration (mg/L) in 100‐fold diluted samples (normal range < 5 mg/L). 4‐PL graph plotting was conducted using GraphPad Prism version 9.4.1 for Windows (GraphPad Software, La Jolla, CA, USA).

#### 2.8.2 Cytokine Profiling

Levels of interleukin (IL)‐1β, IL‐6, IL‐8, IL‐10, and tumor necrosis factor (TNF)‐α in plasma samples were determined using a Magnetic Luminex Performance assay (Human High Sensitivity Cytokine Premixed Kit, R&D Systems Inc.) according to the manufacturer's instructions. Bead fluorescence was measured in triplicate using a Bio‐Plex MAGPIX multiplex reader (Bio‐Rad Laboratories, Hercules, CA, USA). The MDD of each analyte in the assay is presented in **Table** **S2**. A standard curve for each of the five analytes was generated (pg/mL), and a five‐parameter logistic (5‐PL) curve fit was used to calculate the cytokine concentrations in 2‐fold diluted samples. 5‐PL graph plotting was conducted in GraphPad Prism. Cytokine concentrations were log_10_ transformed.

#### 2.8.3 Endotoxin Level Assessment

Plasma endotoxin levels were measured using the Pierce Chromogenic Endotoxin Quant Kit (Thermo Fisher Scientific Inc.), following the manufacturer's protocol. Briefly, plasma samples were serially diluted 10 times with endotoxin‐free water and heat‐shocked (70°C for 10 min). Aliquots (50 µL) of each sample were transferred in duplicate into a 96‐well plate, and 50 µL of the amebocyte lysate reagent was added to each well. The plate was incubated at 37°C for 12 min in a StableTemp plate heater (Cole‐Parmer, Vernon Hills, IL, USA). Then, 100 µL of prewarmed chromogenic substrate solution was added to each well, and the reaction was stopped after incubation for 6 min at 37°C by adding 100 µL of 25% acetic acid. The optical density was immediately measured at 405 nm using an Infinite M200 microplate reader (Tecan, Salzburg, Austria). Endotoxin concentration in each sample was obtained by extrapolation from a standard curve for high standard (0.1–1.0 endotoxin units per mL) generated simultaneously.

#### 2.8.4 TMAO and Precursors Analysis

The levels of TMAO and its precursors were measured in the plasma by UHPLC‐ESI‐MS/MS as previously described [30], with some modifications. In brief, 90 µL plasma was mixed with 900 µL acetonitrile and 10 µL internal standard (isotopically enriched TMAO, d9‐TMAO) and centrifuged at 14 460 × *g* for 10 min at 4°C. Quantitative analysis of the supernatant was performed using a UHPLC DIONEX Ultimate 3000 equipped with a triple quadrupole TSQ Vantage fitted with a heated‐electrospray ionization (H‐ESI II) probe (Thermo Fisher Scientific Inc.). Separation was carried out on an Xbridge BEH HILIC XP (100 × 2.1 mm, 2.5‐µm particle size) (Waters, Milford, MA, USA) using a gradation elution. The elution solvents included acidified water (0.1% v/v formic acid) as solvent A, acetonitrile containing 0.1% v/v formic acid, as solvent B, and a 20 mM ammonium formate solution containing 1% formic acid as solvent C. The gradient started with 10% C, which was maintained constant during the whole analysis, 89% B and 1% A. The mobile phases comprised a program of 0–1 min, 1% A; 1–3 min, to reach 10% A; 3–3.5 min, 10%–43% A, maintained for 2 min; 5.5–6 min, 1% A to return to the starting conditions and maintained until the end of the analysis (10 min). The phases were delivered at a constant flow rate of 0.5 mL/min, the injection volume was 5 µL, and the column temperature was set at 35°C. The mass spectrometer operated in selective reaction monitoring (SRM) mode with positive ionization. The spray voltage was set at 3.5 kV, the vaporizer temperature at 200°C, and the capillary temperature operated at 270°C. The sheath gas pressure was 40 units, and the auxiliary gas pressure was set to five units. Ultra‐high‐purity argon gas was used for the collision‐induced dissociation (CID). The S lens value was 65. Data processing was performed using XCalibur Version 2.1 (Thermo Fisher Scientific Inc.), and quantification was performed using calibration curves built with all available standard compounds.

### 2.9 RNA Extraction, Quantity and Quality Assessment

Total RNA was isolated from frozen PBMC pellets and colonic biopsies. RNA was extracted from PBMCs using the Quick‐RNA Whole Blood Kit (Zymo Research Corporation, Irvine, CA, USA), followed by DNase treatment to avoid DNA contamination, according to the manufacturer's instructions. Colon mucosal biopsies were disrupted in 700 µL of QIAzol Lysis Reagent (Qiagen, Hamburg, Germany) containing phenol and guanidine thiocyanate using a tissue grinder pellet pestle (DWK Life Sciences LLC., Millville, NJ, USA) for 20–40 s. RNA was extracted from the resulting solution using a miRNeasy Mini kit (Qiagen) following the manufacturer's instructions.

RNA yield and purity were evaluated using a NanoDrop ND‐2000 spectrophotometer (Thermo Fisher Scientific Inc.). RNA samples with an absorbance ratio at 260 and 280 nm of ∼2.0 and an absorbance 260/230 ratio between 2 and 2.2 were then run by on‐chip electrophoresis on a 2100 Bioanalyzer system (Agilent Technologies Inc.) using an RNA 6000 Nano kit to assess its quality. All RNA samples scoring an RNA Integrity Number (RIN) of ≥ 8 were stored at –80°C and then considered for further processing.

### 2.10 Quantitative Real‐Time PCR (qPCR) of Intestinal Biopsies

Total RNA (200 ng) from frozen intestinal biopsies was converted into cDNA using the High‐Capacity RNA‐to‐cDNA Kit (Applied Biosystems, Foster City, CA, USA) according to the manufacturer's instructions. For qPCR analysis, synthesized cDNAs were 10‐fold diluted with UltraPure DNase/RNase‐Free Distilled Water (Invitrogen, Waltham, MA, USA), and 3 µL was used with TaqMan Fast Advanced Master Mix (Applied Biosystems) and TaqMan Gene Expression Assay probes (Applied Biosystems). A panel of 7 target genes that may be relevant in IBD etiopathogenesis (*IL‐1β* [31], *IL‐6* [32, 33], *IL‐8* [31, 34], *IL‐12* [35], *IL‐17* [35, 36, 37], interferon γ (*IFN‐γ*) [36, 37], and *TNF‐α* [33, 38]) were selected based on a literature review. Five candidate reference genes (*B2* *M*, β‐2‐microglobulin; *GAPDH*, glyceraldehyde‐3‐phosphate dehydrogenase; *HPRT*, hypoxanthine‐guanine phosphoribosyl transferase; *PPIA*, peptidylprolyl isomerase A; *RPLP0*, ribosomal protein lateral stalk subunit P0) were selected from the literature [38, 39, 40]. All TaqMan probe sets used in this study were purchased from Thermo Fisher Scientific (**Table** **S3**). qPCR was performed in triplicate using a CFX Connect Real‐Time PCR Detection System (Bio‐Rad Laboratories). The thermal cycler conditions were as follows: 2 min at 50°C and 2 min at 95°C, followed by 40 cycles at 95°C for 3 s and 60°C for 30 s. Among candidate reference genes, *HPRT*, *PPIA*, and *RPLP0* genes were identified as the most stable reference genes, with M values < 0.5, and used to normalize the expression of the target genes with the 2^−ΔΔCq^ method. Data were processed using Bio‐Rad CFX Maestro 2.3 Version 5.3.

### 2.11 Identification and Quantification of Pomegranate ET‐Derived Metabolites in Urine and Plasma and Subject Stratification into Urolithin Metabotypes

Urine samples were defrosted and processed as previously described [41]. Briefly, urine samples were vortexed, diluted in 0.1% formic acid in water (1:4, v/v), vortexed again, centrifuged at 14 460 × *g* for 5 min, and supernatants were filtered (0.22‐µm nylon filter) before the UHPLC‐ESI‐MS/MS analysis.

Plasma samples were defrosted and extracted, as previously described [42]. Briefly, 400 µL aliquots were mixed with 1 mL of 2% formic acid in acetonitrile. The samples were vortexed and ultrasonicated for 10 min. After centrifugation at 14 460 × *g* for 10 min, supernatants were reduced to dryness under vacuum using a SpeedVac concentrator (Thermo Scientific Inc.) and resuspended in 100 µL of methanol:water:formic acid (50:50:0.1, v/v/v), centrifuged again at 14 460 × *g* for 10 min, and placed into vials for UHPLC‐ESI‐MS/MS analysis.

Urine and plasma samples were analyzed according to the method reported by Ludwig et al. (2015) [42], with some modifications. UHPLC‐ESI‐MS/MS analyses were performed using a UHPLC DIONEX Ultimate 3000 equipped with a TSQ Vantage triple quadrupole MS fitted with heated ESI (Thermo Scientific Inc.). Separations were carried out using a Kinetex EVO C18 (100 × 2.1 mm), 2.6 µm particle size (Phenomenex, Torrance, CA, USA), installed with a precolumn cartridge (Phenomenex). For UHPLC, mobile phase A was 0.1% formic acid in water, and mobile phase B was acetonitrile containing 0.1% formic acid. The gradient started with 5% phase B, and isocratic conditions were maintained for 0.5 min. Eluent B reached 75% after 7.5 min, and the gradient was maintained for 2 min prior to re‐establishing the starting conditions, which were maintained for 3 min to re‐equilibrate the columns. The flow rate was 0.4 mL/min, the injection volume was 5 µL, and the column temperature was set at 40°C. Target precursor ions and characteristic product ions were monitored in SRM mode using negative ionization. The spray voltage was set at 3 kV, the vaporizer temperature at 300°C, and the capillary temperature operated at 270°C. The sheath gas flow and auxiliary gas pressure were set to 50 and 10 units, respectively. Ultrahigh‐purity argon gas was used for the CID. The S‐lens values were defined for each compound based on infusion parameter optimization or by using the values obtained for the chemically closest available standards for compounds that were not available for infusion (**Table** **S4**). Quantification was performed using calibration curves of standards when available or using the most structurally similar compound. Data were processed using XCalibur Version 2.1 (Thermo Fisher Scientific Inc.).

The allocation of subjects into urolithin metabotypes based on the selective production of urolithins was performed following the available literature [13]. Briefly, subjects were classified into the following metabotypes:

- Metabotype 0: no urolithins detected in urine;
- Metabotype A: urolithin A‐glucuronide found in urine, but no urolithin B or isourolithin A derivatives detected;
- Metabotype B: urolithin B‐glucuronide or isourolithin A‐glucuronide found in urine, regardless of the presence of urolithin A derivatives.

### 2.12 Microarray‐Based Transcriptome Analysis of PBMCs

Microarray analysis was carried out on Agilent Human Gene Expression Microarrays (072363) using a Low Input Quick Amp Labeling Kit and a One‐Color RNA Spike‐In Kit, following the One‐Color Microarray‐Based Gene Expression Analysis protocol from Agilent Technologies. Briefly, experimental RNA (200 ng) in the presence of an external RNA spike‐in mixture was labeled with the fluorescent dye Cyanine3 (Cy3) after reverse transcription and amplification. Cy3‐labeled cRNA was purified using an RNeasy Mini Kit (Qiagen). cRNA yield (µg) and specific activity (pmol Cy3 per µg cRNA) were assessed using the Micro Array measurement tab of a NanoDrop ND‐2000 spectrophotometer (Thermo Fisher Scientific Inc.). Six hundred nanograms of Cy3‐labeled cRNA (specific activity > 6.0 pmol Cy3/µg cRNA) was fragmented at 60°C for 30 minutes following the manufacturer's instructions. Then, hybridization was performed for 17 h at 65°C at 10 rpm in a rotating hybridization oven (Agilent Technologies Inc.). Microarrays were subsequently washed according to the Agilent Gene Expression Hybridization protocol. Using a one‐color scan setting, the slides were immediately scanned using an Agilent G2565AA Microarray Scanner System. Microarray images were processed using the Feature Extraction Software (Version 12.0; Agilent Technologies Inc.) with default parameters (GE1_1200_Jun14 and Grid:072363_D_F_20150612) to obtain the features for data analysis.

### 2.13 Differential Gene Expression and Functional Analyses of PBMCs

Principal component analysis (PCA) of the PBMC expression profiles was conducted using *PCAtools* R package (https://github.com/kevinblighe/PCAtools) and visualized using the *biplot* function in the *ggplot2* R package [43]. Differential expression in PBMCs was determined using a paired‐sample *t*‐test in GeneSpring GX version 11.5 software package (Agilent Technologies Inc.) to test for differences between gene expression levels at baseline (*t*_0_) and at the end of the study (*t*_1_) in the POMJ and placebo groups. Differentially expressed genes (DEGs) were identified where there was at least a 0.5 log_2_‐fold‐change in intensity values and a *p* value ≤ 0.01. Functional analysis was performed using the “Canonical Pathway Analysis” and “Diseases and Biofunctions” modules of ingenuity pathway analysis (IPA) (Qiagen). The −log (*p* value) ≥ 2 was taken as the threshold. The algorithm used to calculate the *Z*‐scores and *p* values of the overlap has been described previously [44]. Heatmaps of the gene expression levels were generated using the *pheatmap* package in R (https://CRAN.R-project.org/package=pheatmap). To validate the microarray results, four genes (*ATF2*, activating transcription factor 2; *HSPA14*, heat shock protein family A (Hsp70) member 14; *PMS1*, PMS1 homolog 1; *MTIF3*, mitochondrial translational initiation factor 3) were selected for qPCR analysis using an unbiased, random selection approach. Additional details are provided in the Supporting Information.

### 2.14 Statistical Analyses

Nutritional profiles between POMJ and placebo were compared using Euclidean distance and Pearson correlation coefficient (calculated using Microsoft Excel version 16) to assess overall nutrient differences and pattern matching, respectively.

Baseline characteristics and other assessments are summarized as means ± standard deviation (SD), median (interquartile range, IQR), or *n* (%). Levels of FC, TMAO, and its precursors in plasma; ET metabolites in plasma and urine; and quantitative gene expression analysis by qPCR were compared using a two‐tailed, ratio‐paired *t*‐test in the two groups before and after the intervention. These data were analyzed using GraphPad Prism version 10.3.0 for Windows (GraphPad Software, La Jolla, CA, USA). For these analyses, a *p* value less than 0.05 was considered statistically significant.

To describe the inflammatory outcomes with significant post‐intervention variation, a probability density function (PDF) was generated through kernel density estimation (KDE) to evaluate differences in distribution between CD and UC. KDE was performed using the Python programming language, custom scripts, and the *sklearn* package [45]. The Spearman correlation was used to assess associations between the ratio *t*₁/*t*₀ of the inflammatory outcomes with significant post‐intervention variation and the ratio *t*₁/*t*₀ of urinary urolithins using GraphPad Prism.

## 3 Results

### 3.1 Nutritional and (Poly)Phenol Content of Experimental Beverages

The POMJ and placebo beverage had the same nutritional profile (**Table** **S5**), as evidenced by minimal differences (Euclidean distance: 4.70) and matching patterns (Pearson correlation coefficient: 1.00), except for the (poly)phenol content of POMJ (**Table** **S6**). One serving (125 mL) of POMJ made from arils and whole fruit with peels provided 450 mg of (poly)phenols, of which approximately 80% was ETs. The measured ET concentrations, along with ellagic acid and its derivatives (**Table** **S6**), were comparable to those reported for the same pomegranate cultivar used here, Wonderful [46, 47, 48], resulting in a daily POMJ serving (twice a day) that provided more than 700 mg of ETs—several times higher than the average daily intake in European populations (5–12 mg/day) [49, 50].

### 3.2 Enrollment of Study Participants

The flow of participants for the study is shown in **Figure** **S1**. Among the screened patients diagnosed with IBD between January 2017 and September 2018, eighteen subjects (11 males and 7 females) were successfully enrolled; two patients in the POMJ group did not complete the study. In total, 16 volunteers completed the study (POMJ: 5 males and 5 females; placebo: 5 males and 1 female). No serious adverse events occurred during the study period. This study was conducted in compliance with the CONSORT (Consolidated Standards of Reporting Trials) guidelines for reporting parallel‐group randomized trials [51].

**Table** **1** summarizes the demographic and clinical characteristics of participants. Six subjects had CD, and 12 had UC. Dietary TAC has been related to several health outcomes [52]; values evaluated as FRAP through the FFQ were consistent with those of other studies in healthy or unhealthy Italian subjects [28, 53].

**TABLE 1:** Baseline characteristics of the study participants.

Baseline characteristics	*n* (%)	Mean (SD; range)
Age (years)		56 (12; 30–71)
Sex:		
Female	6 (38%)	
Male	10 (62%)	
Ethnicity:		
Caucasian	16 (100%)	
BMI (kg/m^2^):		25 (4; 19–34)
Underweight	0 (0%)	
Normal weight	7 (44%)	
Overweight	7 (44%)	
Obese	2 (12%)	
Type of disease:		
CD	6 (38%)	
UC	10 (62%)	
Ongoing treatment:		
Mesalamine	9 (56%)	
Mesalamine + Azathioprine	1 (6%)	
Anti–TNF‐α	2 (13%)	
Mesalamine + Anti–TNF‐α	1 (6%)	
None	3 (19%)	
Pomegranate juice consumption:		
Regularly	1 (6%)	
Occasionally	2 (13%)	
Never	13 (81%)	
Dietary total antioxidant capacity as FFQ‐based FRAP (mmol Fe/kg)		20 (12; 7–50)

Abbreviations: BMI: Body Mass Index; CD: Crohn's disease; FFQ: food‐frequency questionnaire; FRAP: ferric reducing antioxidant power; TNF‐α: tumor necrosis factor α; UC: ulcerative colitis.

### 3.3 Levels of the Fecal Marker of Gut Inflammation

**FIGURE 2:**
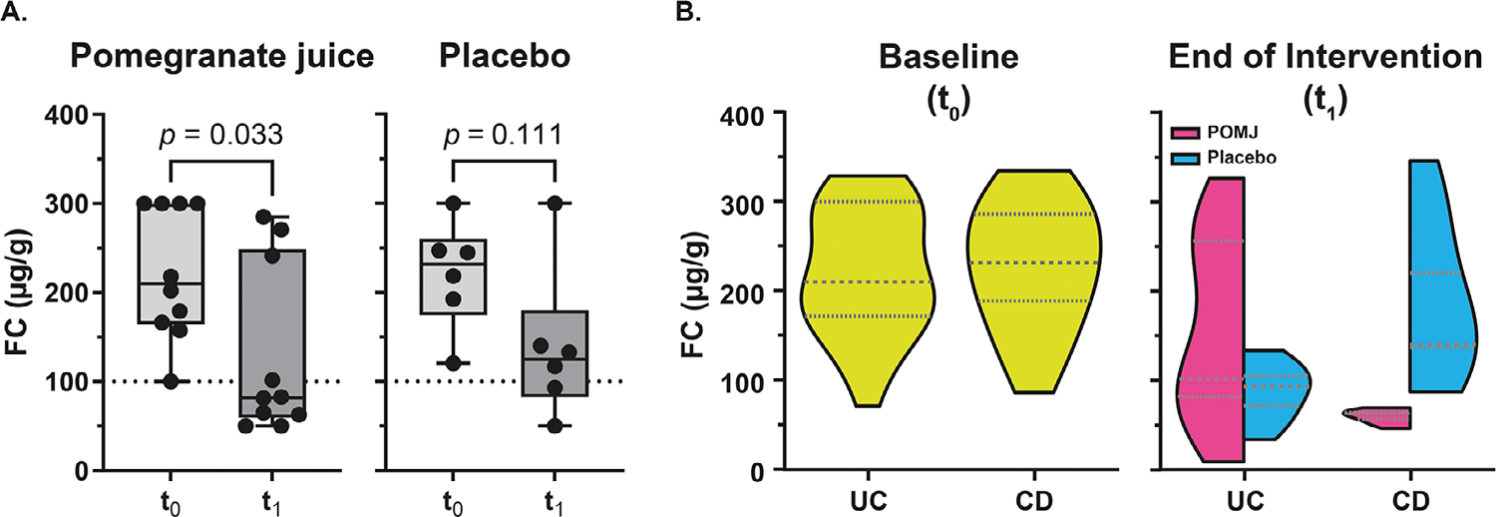
Fecal calprotectin (FC) levels in patients with IBD in clinical remission at baseline (*t*_0_) and at the end of the intervention (*t*_1_). (A) FC levels in patients who received (left) pomegranate juice or (right) placebo. The box represents the lower and upper quartiles, and the horizontal line in the middle of the box denotes the median. The reference limit for a raised value is 100 µg/g. *p* values were reported from comparisons by ratio‐paired *t*‐test. (B) Probability density distributions of FC levels at (left) baseline (*t*_0_) and (right) the end of intervention (*t*_1_) with pomegranate juice or placebo. The violin plots present FC distributions in different patient groups, ulcerative colitis (UC) or Crohn's disease (CD), obtained by a kernel density estimator (KDE), with the median (the dashed bar in the center of the bar) and the interquartile range (the dotted bars representing the 25^th^ and 75^th^ percentiles).

Compared to baseline (*t*_0_), FC decreased approximately 2.4‐fold (*p* value  =  0.033) after intervention with POMJ (*t*_1_), whereas no significant changes were observed in the placebo group (*p* value  =  0.111) (**Figure** **2A**).

The identification of patients at increased risk of relapse is based on FC levels, which vary between studies (50 to 340 µg/g) depending on disease type and phenotype [54]. In this study, we used an FC level ≥ 100 µg/g as the threshold, which identifies patients in clinical remission with a significant likelihood of relapse [55, 56, 57]. While FC levels did not indicate relapse in any subjects, the POMJ group showed better maintenance of remission compared to the placebo group.

The distribution of the FC levels at baseline and 12 weeks after the intervention is depicted in violin plots in **Figure** **2B**. The distribution of FC levels was similar in patients with CD and patients with UC at baseline. After 12 weeks of intervention, the FC levels in the POMJ group were significantly lower in subjects with CD (good responders) than in those with UC (poor or moderate responders).

### 3.4 Evaluation of Disease Activity Scores

The MES at baseline and after 12 weeks of intervention was not significantly different in the POMJ and placebo groups (**Table** **S7**). Regarding the effect of the intervention on disease activity scores, we found no significant pre‐ to post‐intervention changes in the SCCAI for patients with UC or in the CDAI for patients with CD in either study group (**Table** **S7**). Overall, no subjects relapsed (active disease, defined as SCCAI > 5 or CDAI > 150 [23, 24, 25]) after either intervention.

### 3.5 Serological Levels of Inflammatory Markers, Endotoxins, and Plasma Levels of TMAO and Its Precursors

**FIGURE 3:**
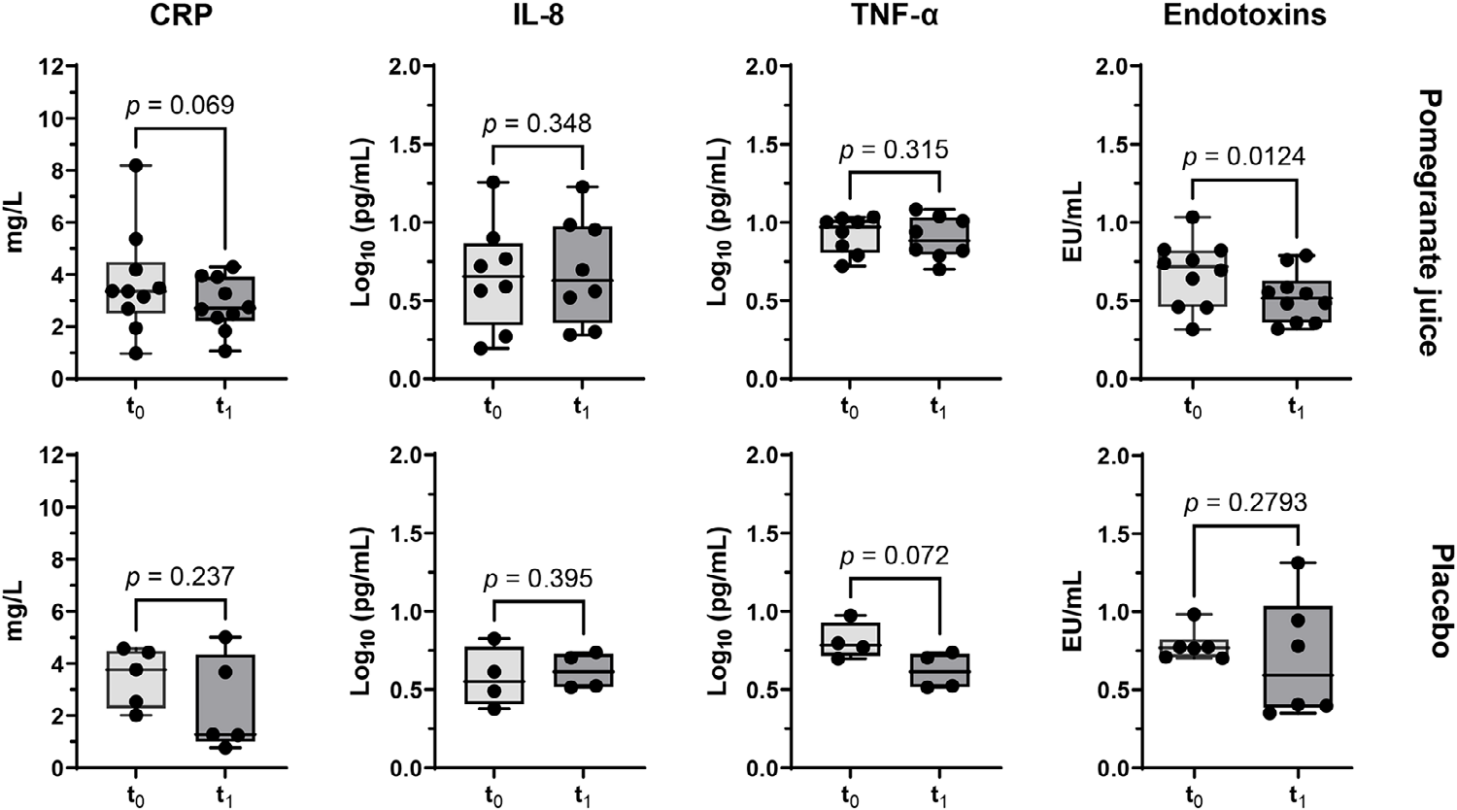
Circulating levels of CRP, cytokines (IL‐8 and TNF‐α), and endotoxins at baseline (*t*_0_) and 12 weeks after intervention (*t*_1_) with pomegranate juice or placebo in patients with IBD in clinical remission. Cytokine concentrations were log_10_‐transformed. *p* values were reported from comparisons by ratio‐paired *t*‐test. CRP: plasma C‐reactive protein; EU: endotoxin units; IL: interleukin; TNF‐α: tumor necrosis factor.

CRP, an important modulator of inflammatory and immune responses [58], showed a slight decrease over the 12‐week study period, but the changes were not statistically significant (**Figure** **3**). This is consistent with the tendency of CRP levels to normalize in stable, quiescent IBD, approaching levels similar to those in healthy subjects (0–7 mg/L) [58, 59].

Compared to baseline, there were no significant changes in CRP and other serological markers of inflammation, such as IL‐8 and TNF‐α, in the patients after intervention with POMJ or placebo (**Figure** **3**). Levels of IL‐1β, IL‐6, and IL‐10 in most pre‐ and post‐treatment samples were found to be below the detection range in the ELISA assay and were not analyzed further. Plasma levels of TMAO and its precursors (**Table** **S8**) remained unchanged after both interventions compared to those at baseline (*t*_0_), except for betaine, which increased in the placebo group. In contrast, plasma endotoxin levels were reduced in the POMJ group compared to baseline (**Figure** **3**), with UC patients showing a greater but more variable reduction, whereas CD patients showed a more uniform but less pronounced response (**Figure** **S2**).

### 3.6 Measurement of Cytokine Gene Expression in Colonic Mucosa

There were no significant differences in the expression of the selected genes (IL‐1β, IL‐6, IL‐8, IL‐12, IL‐17, IFN‐γ, and TNF‐α) in the colon mucosal biopsy after the intervention in comparison with baseline (**Figure** **S3**). This is consistent with the high degree of variability in gene expression data obtained from colon tissues, as reported previously [60].

### 3.7 ET‐Metabolite Profiling and Quantification

Up to 15 different ET metabolites, including 14 urolithins (the main ET gut microbiota derivatives) and dimethylellagic acid‐glucuronide, were targeted by UHPLC‐ESI‐MS/MS (**Table** **S4**). All compounds detected and quantified in the plasma (**Table** **S9**) and urine (**Table** **2**) were phase II conjugates of urolithin B, urolithin A, and urolithin C (sulfates and glucuronides) or ellagic acid (dimethylellagic acid‐glucuronide) and showed high inter‐individual variation. POMJ consumption led to an increase in the urinary concentrations of urolithin B‐glucuronide, urolithin B‐sulfate, isourolithin A‐sulfate, and total urolithins (**Table** **2**). The ratio of isourolithin A‐sulfate (IsoUroA‐S) between the end of the intervention and the baseline (*t*₁/*t*₀) showed a strong positive correlation with the corresponding ratio of FC levels (Spearman correlation coefficient *ρ* = 0.8) (**Figure** **S4**). In the placebo group, urolithin concentrations in the urine remained low, and total urolithin levels did not increase significantly during the intervention.

**TABLE 2:** Metabolites characterized and quantified in urine at baseline (*t*_0_) and 12 weeks after intervention (*t*_1_) with pomegranate juice (POMJ) or placebo in patients with IBD in clinical remission.

Urolithin (µM)	Median (IQR) POMJ *t*_0_ ^a^	Median (IQR) POMJ *t*_1_ ^a^	*p* value	Median (IQR) Placebo *t*_0_	Median (IQR) Placebo *t*_1_	*p* value
UroB‐GlcUA^b^	0.195 (0.095–3.405)	5.093 (0.320–24.828)	**0.0467**	0.102 (0.080–0.129)	0.215 (0.152–0.265)	**0.0224**
UroB‐S	0.014 (0.004–0.017)	0.027 (0.022–0.053)	**0.0029**	0.004 (0.002–0.005)	0.006 (0.003–0.010)	0.4061
UroA‐GlcUA^b^	0.232 (0.064–4.712)	14.247 (2.638–19.082)	0.0991	0.000 (0.000–0.031)	0.148 (0.036–0.667)	> 0.9999
IsoUroA‐GlcUA^b^	0.042 (0.020–1.571)	8.072 (0.036–9.912)	0.0720	0.000 (0.000–0.009)	0.000 (0.000–0.010)	> 0.9999
IsoUroA‐S	0.006 (0.001–0.007)	0.018 (0.008–0.022)	**0.0282**	0.002 (0.000–0.005)	0.006 (0.004–0.007)	0.6673
IsoUroA‐S‐GlcUA	0.046 (0.012–0.059)	0.088 (0.050–0.112)	0.2201	0.000 (0.000–0.023)	0.000 (0.000–0.031)	> 0.9999
UroC‐GlcUA	0.000 (0.000–0.000)	0.000 (0.000–0.000)	> 0.9999	0.017 (0.000–0.123)	0.000 (0.000–0.115)	0.4944
UroC‐S	0.006 (0.003–0.014)	0.012 (0.009–0.037)	0.0920	0.013 (0.003–0.015)	0.014 (0.000–0.037)	0.1633
DMEAG	0.000 (0.000–0.269)	0.146 (0.054–0.516)	0.3369	0.000 (0.000–0.000)	0.020 (0.000–0.054)	> 0.9999
Total urolithins	0.428 (0.321–9.669)	27.659 (3.129–68.553)	**0.0356**	0.136 (0.094–0.183)	0.481 (0.291–0.797)	0.0549

Note: *p* values were reported from comparisons by ratio‐paired *t*‐test; significant values (*p* < 0.05) are marked in bold.

^a^ Three subjects were excluded from the analysis because urine samples were not available for both *t*_0_ and *t*_1_. Zero values were excluded from statistical analysis.

^b^ Compounds used for metabotyping.

Abbreviations: DMEAG: dimethylellagic acid‐glucuronide; IQR: interquartile range (Q1 to Q3); IsoUroA‐GlcUA: isourolithin A‐ glucuronide; IsoUroA‐S: isourolithin A‐sulfate; IsoUroA‐S‐GlcUA: isourolithin A‐sulfate‐glucuronide; UroA‐GlcUA: urolithin A‐glucuronide; UroB‐GlcUA: urolithin B‐glucuronide; UroB‐S: urolithin B‐sulfate; UroC‐GlcUA: urolithin C‐glucuronide; UroC‐S: urolithin C‐sulfate.

The plasma concentrations of urolithins were very low and did not change in either the POMJ or placebo group (**Table** **S9**). Based on clustering according to urolithin production [13], most volunteers belonged to metabotype B at *t*_0_, while all of them were allocated to metabotype B at the end of the intervention (**Table** **S10**). In general, metabotype allocation was well preserved after the 12‐week intervention period.

### 3.8 Genome‐Wide Differential Gene Expression Analysis in PBMCs

**FIGURE 4:**
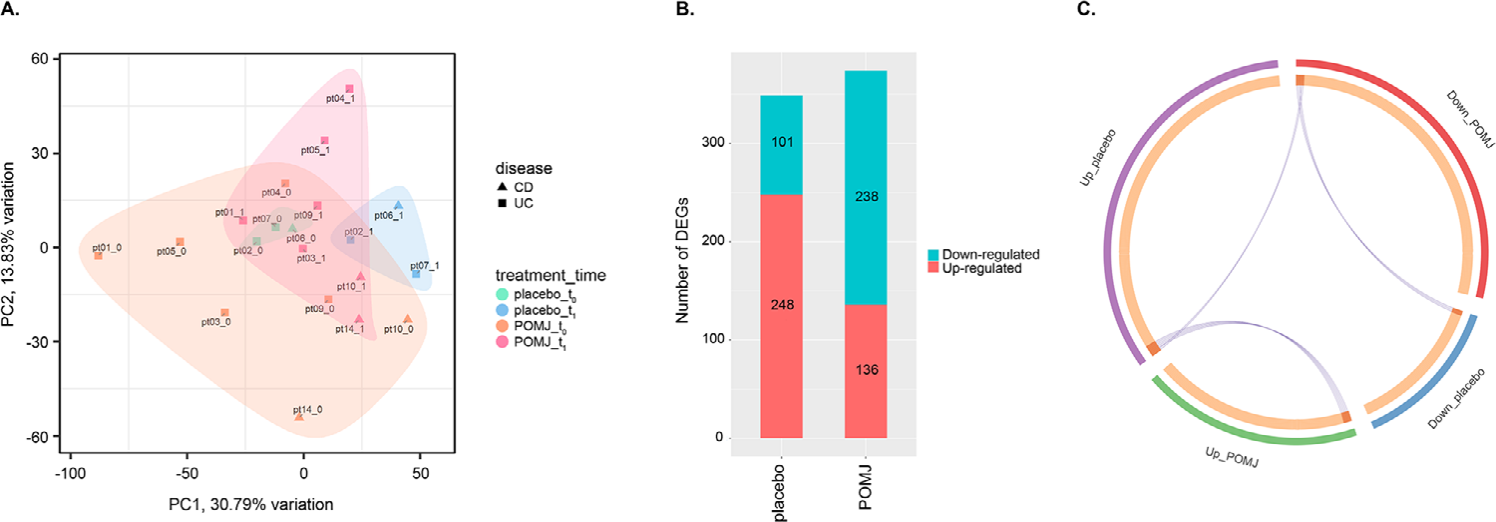
Gene expression analysis of peripheral blood mononuclear cells (PBMCs) of patients with IBD in clinical remission receiving either pomegranate juice (POMJ) or placebo for 12 weeks. (A) Principal component analysis of gene expression profiles of PBMCs from patients with UC (ulcerative colitis) or CD (Crohn's disease) treated with POMJ or placebo, at baseline (*t*_0_) and at the end of the intervention (*t*_1_). 0.1% of variables were removed based on low variance. (B) Number of up‐ or downregulated genes in PBMCs after 12‐week intervention with POMJ or placebo (log_2_ fold‐change ≥ 0.5 or ≤ −0.5, *p* value ≤ 0.01), compared to baseline. (C) Overlap between up or downregulated genes in POMJ and placebo groups. In the circus plot, each arc on the outside represents a gene list. On the inside, each gene member of that list is assigned a spot on the arc. The dark orange color represents the genes shared by multiple lists and the light orange color represents genes unique to that gene list. Purple lines link genes that are shared by multiple gene lists. DEGs: differentially expressed genes; PC: principal component.

PCA of the microarray data revealed a shift in PBMC expression profiles over the 12‐week intervention period for all patients (**Figure** **4A**). While there was no clear separation between POMJ and placebo groups, patients receiving POMJ showed greater variance and generally more pronounced changes from *t*_0_ to *t*_1_. No differential clustering by disease type was observed.

Comparison of transcript intensity values between *t*_0_ and *t*_1_ identified 349 and 374 DEGs (log_2_ fold‐change ≥ 0.5 or ≤ −0.5, *p* value ≤ 0.01) after POMJ or placebo treatment, respectively (**Figure** **4B**). Very few genes were differentially expressed in both intervention groups (**Figure** **4C**).

qPCR analysis of four independently selected genes (*ATF2*, *HSPA14*, *MTIF3*, and *PMS1*) largely confirmed the microarray results. In the POMJ group, PMS1 was significantly modulated at *t*_1_ versus *t*_0_ in both analyses, while the other genes showed weak but consistent downregulation. The placebo group showed no significant modulation of these genes (**Table** **S11**).

### 3.9 POMJ‐ and Placebo‐Associated Pathway Modulation in PBMCs

**FIGURE 5:**
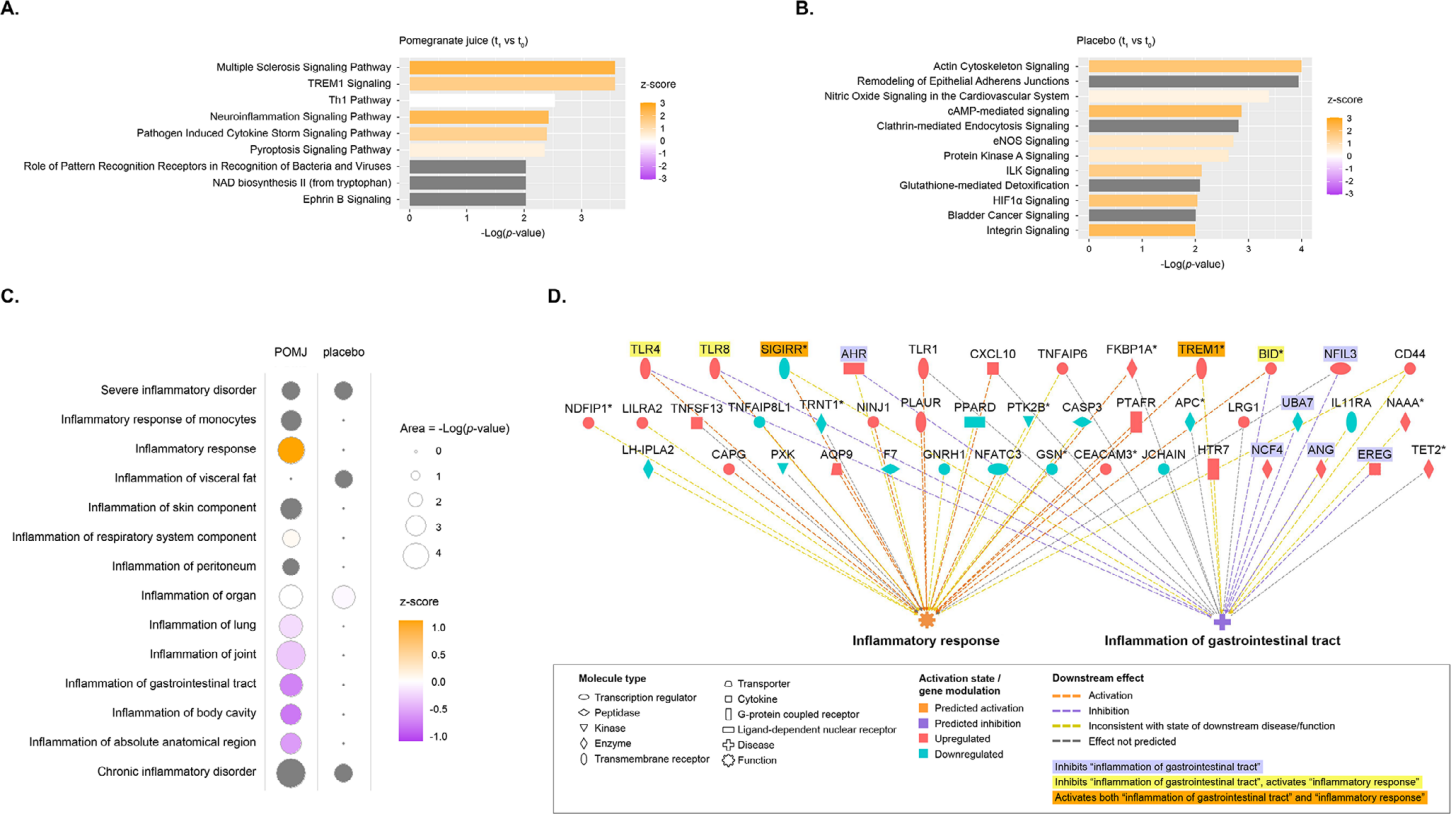
Functional analysis of differentially expressed genes (DEGs) in peripheral blood mononuclear cells of patients with IBD in clinical remission after a 12‐week intervention involving consumption of pomegranate juice (POMJ) or placebo, compared to baseline. (A) Significantly altered canonical pathways (−Log *p* value ≥ 2) in the POMJ group. A positive *z*‐score denotes pathway activation, and a negative z‐score denotes pathway inhibition. Grey bars represent a pathway with no activity pattern available. (B) Same as in (A), but for the placebo group. (C) Predicted effects (activation‐inhibition) of DEGs on downstream biological functions belonging to functional categories “inflammation,” “inflammatory response,” “inflammatory disorder,” and “chronic inflammatory disorder,” in the POMJ and placebo group. Terms with *p* value ≤ 0.01 in at least one of the two study groups are reported. Grey circles indicate that no activity pattern is available for the prediction of function activation state. (D) Mechanistic networks reporting molecules up‐ or downregulated in the POMJ group and their interactions with the downstream functions “inflammatory response” and “inflammation of gastrointestinal tract.”.

Canonical pathway analysis of DEGs revealed distinct modulations in PBMCs after 12 weeks of POMJ or placebo supplementation. In the POMJ group, pathways involved in immune and inflammatory responses were predominantly enriched (**Figure** **5A**). Notable activated pathways included multiple sclerosis, triggering receptor expressed on myeloid cells‐1 (TREM1), pathogen‐induced cytokine storm, and pyroptosis signaling.

In contrast, the placebo group showed significant modulation in pathways related to cytoskeleton organization, signal transduction, and oxygen‐sensing and nitric oxide (NO)‐mediated pathways (**Figure** **5B**). These included activation of actin cytoskeleton signaling, integrin‐linked kinase (ILK) signaling, cAMP‐mediated signaling, and HIF1α signaling.

Interestingly, while genes participating in these pathways were mostly upregulated at *t*_1_ compared to *t*_0_ in the placebo group, a similar trend was observed in some patients who received POMJ, although the differences remained below the thresholds for differential expression (**Figure** **S5**)

### 3.10 POMJ Downstream Effects on Inflammation

IPA‐based “Diseases and Biofunctions” analysis was performed to infer the impact of DEGs on downstream biological functions related to inflammation in individuals with IBD receiving POMJ or placebo.

In the POMJ group, the inflammatory response was predicted to be activated (*z*‐score = 1.12, *p* value = 5.91E−05), consistent with the canonical pathway analysis results. However, inflammation of the body cavity (*z*‐score = −0.81, *p* value = 5.88E−04) and inflammation of the GI tract (*z*‐score = −0.75, *p* value = 2.40E−04) were predicted to be suppressed. These functions were not significantly altered in the placebo group between *t*_0_ and *t*_1_ (**Figure** **5C**).

Analysis of the mechanistic networks (**Figure** **5D**) revealed complex interactions between DEGs and downstream inflammatory functions. Two genes, *SIGIRR* (single Ig and TIR domain containing) and *TREM1* (triggering receptor expressed on myeloid cells 1), were predicted to activate both the inflammatory response and inflammation of the GI tract. *SIGIRR*, a negative regulator of TLR and IL‐1R signaling pathways, was downregulated, while *TREM1*, an inflammation amplifier, was upregulated.

Three upregulated genes—*BID* (BH3 Interacting Domain Death Agonist), *TLR4*, and *TLR8* (Toll‐like receptors 4 and 8)—were predicted to increase the overall inflammatory response while reducing inflammation of the GI tract.

Several genes were associated with decreased GI tract inflammation, including *AHR* (aryl hydrocarbon receptor), *ANG* (ribonuclease angiogenin), *EREG* (epiregulin), *NCF4* (neutrophil cytosolic factor 4), *NFIL3* (nuclear factor interleukin 3 regulated), and *UBA7* (ubiquitin‐like modifier activating enzyme 7). The upregulation of *AHR*, *ANG*, *EREG*, *NCF4*, and *NFIL3* and downregulation of *UBA7* contributed to this effect. Overall, individuals assigned to the POMJ group showed more pronounced modulation of genes upstream of the inflammatory response or inflammation in the GI tract compared to those receiving placebo (**Figure** **S6**).

## 4 Discussion

The hypothesis that consumption of POMJ for 12 weeks by patients with IBD in clinical remission with a high risk of relapse would exert anti‐inflammatory effects at both the local (colon) and systemic levels was only partially supported by the results of this study. At the end of the intervention, the primary outcome measure, FC levels, and one of the secondary outcomes, plasma endotoxin levels, were significantly lower in patients who received POMJ, whereas no changes were observed in the placebo group. However, some of the secondary outcomes, including the endoscopic markers of mucosal inflammation, colonic mucosal cytokine expression, and serological markers of inflammation such as CRP, IL‐8, TNF‐α, TMAO, and most of its precursors, were not modified by either POMJ or placebo intake. This may indicate a stable condition (no patient relapsed during the intervention); however, it also did not show a marked effect of POMJ supplementation over the placebo.

It is established that stool biomarkers such as FC are more accurate than other serum inflammatory markers, such as CRP, in detecting subclinical inflammation and predicting possible disease relapse in patients in remission with CD [61] and UC [62]. Our results demonstrate that endotoxin levels could also be part of a panel of biomarkers to monitor patients for risk of relapse. Endotoxins are lipopolysaccharides, a component of the cell membrane of Gram‐negative bacteria that can be translocated from the intestinal lumen into the bloodstream. Systemic endotoxemia occurs when the intestinal mucosa is damaged during clinical relapse and has been found to correlate positively with disease activity [63, 64]. Therefore, FC and plasma endotoxin levels are effective tools for identifying inactive diseases with significant ongoing inflammation, a setting compatible with the risk of flare.

Although most peripheral inflammatory markers remained largely unchanged, with the notable exception of decreased endotoxins, transcriptomic analysis of PBMCs revealed alterations in the inflammatory response in patients receiving POMJ, but not in the placebo group. This discrepancy highlights PBMC gene expression as a sensitive biomarker for nutritional interventions [65], capable of capturing early inflammatory modulations before they are detectable in circulating proteins. PBMCs play a dual role in IBD pathophysiology—they both contribute to intestinal inflammation and serve as indicators of disease activity and treatment response [66, 67]. Notably, many dysregulated genes in PBMCs overlap with those found in intestinal mucosa samples [66, 68], making peripheral blood transcriptomics a valuable surrogate for intestinal inflammatory status.

While the expression of some genes indicated increased systemic inflammation, others suggested an attenuation of inflammation in the GI tract. This differential response may be attributed to the production of bioactive metabolites, primarily urolithins, from POMJ ETs in the gut. These phenolic metabolites are likely responsible for the observed effects in vivo, given their enhanced bioavailability and reduced elimination compared to their parent compounds.

Particularly, AHR, a ligand‐dependent transcription factor broadly expressed in immune and non‐immune cells in the gut, can be modulated by a wide range of endogenous and exogenous ligands, including urolithins [69, 70]. *AHR* has been reported to be downregulated in inflamed mucosal samples from patients with IBD [71], suggesting its potential as a therapeutic target for immune‐mediated diseases [72]. In this context, the *AHR* upregulation observed in our study is particularly noteworthy given its emerging role in IBD pathophysiology. AHR activation has been associated with multiple beneficial effects in IBD, including modulation of immune responses [73], regulation of inflammation via NF‐κB signaling inhibition [74], and attenuation of cytokine‐induced inflammatory signaling [75]. Furthermore, it enhances gut barrier function through upregulation of tight junction proteins [76], thereby reducing microbial translocation [77], and exerts positive influences on gut microbiome composition [78]. These AHR‐mediated effects align closely with our observations of reduced endotoxin and FC levels, providing a plausible mechanistic link between POMJ consumption and reduced intestinal inflammation. The upregulation of *AHR* in our study, contrasting with its typical downregulation in IBD, may represent a key mechanism by which POMJ exerts its beneficial effects.

Other transcriptomic changes observed in PBMCs provide insights into POMJ's systemic effects on other inflammatory pathways, particularly genes associated with mucosal immunity and gut homeostasis, several of which have been previously linked to IBD. Upregulation of genes such as *NCF4* and *NFIL3* suggests improvements in pathogen clearance and mucosal immunity regulation. *NCF4* contributes to gut microbial composition and antimicrobial activity [79], while NFIL3 plays a role in regulating innate lymphoid cells [80, 81]. Other upregulated genes, including *UBA7* [82] and *ANG* [83, 84], have been demonstrated to play roles in shaping gut microbial composition and maintaining gut health, often acting as antimicrobial peptides [85, 86].

The POMJ intervention also led to the upregulation of three critical genes involved in the inflammatory response: *BID*, *TLR4*, and *TLR8*. *BID*, a member of the BCL2 family, has been shown to play a protective role in IBD by maintaining epithelial integrity and resolving inflammation [87]. TLR4 and TLR8, both members of the TLR family, are fundamental in pathogen recognition and activation of innate immunity. Notably, knockout studies of *BID* and *TLR8* in mice resulted in increased colitis and intestinal inflammation [87, 88], supporting their association with decreased local inflammation as suggested by our IPA analysis.

These molecular changes may collectively enhance epithelial barrier function and regulate mucosal immune response, therefore explaining the observed reduction in FC levels and linking POMJ consumption to reduced intestinal inflammation in patients with IBD. The specific genes modulated in the PBMC transcriptome have established roles in intestinal homeostasis: enhancing barrier function through upregulation of tight junction proteins and reducing microbial translocation (AHR; [76, 77]), controlling innate lymphoid cell development (NFIL3; [80]) and regulating host‐microbiome interactions (NCF4; [79]) crucial for mucosal homeostasis. The significance of these transcriptomic changes is supported by functional evidence, such as the reduction of plasma endotoxin levels and the 2.4‐fold decrease in FC levels.

Unlike the placebo group, in the POMJ group, two main pathways, actin‐cytoskeleton/integrin pathways and NO/HIF‐1α signaling, were not activated. Actin‐cytoskeleton and integrin pathways are crucial for intestinal epithelial integrity and mucosal permeability, and their dysregulation is linked to IBD pathogenesis [89, 90]. NO and HIF‐1α signaling play complex roles in IBD, with excessive activation potentially leading to oxidative stress and pathological angiogenesis [91, 92]. The absence of activation in these pathways in the active treatment group suggests that ET‐derived metabolites may help regulate these processes, potentially contributing to its beneficial effects in IBD.

Differing from PBMC results, no significant changes in the expression of key pro‐inflammatory cytokines and signaling molecules were observed in colonic biopsies. This highlights the complex nature of inflammatory responses in IBD [93], which can differ markedly between intestinal mucosa and systemic circulation and could be attributed to several factors. First, the distinct cellular composition and niche‐specific gene expression patterns of colonic tissue versus PBMCs [94], varying sensitivities to ET‐derived metabolites of both tissues due to mucosal host‐microbe interactions [95], and differences in the temporal dynamics of gene expression changes between these two compartments [96]. Moreover, the selected panel of genes for colonic biopsy analysis may not have captured the full spectrum of local inflammatory modulation, whereas the unbiased whole transcriptome approach in PBMCs revealed a broader range of affected pathways. This observation underscores the importance of considering multiple tissue types and analytical approaches when evaluating the effects of nutritional interventions in complex diseases like IBD.

One of the most interesting aspects of this study is the contrast between the reduction of local inflammation in the GI tract and the increase of systemic inflammatory markers in PBMCs. We speculate that this apparent contradiction may represent a rebalancing of the immune system toward a more regulated state. Indeed, locally at the GI level, the anti‐inflammatory effect may be related to changes in urolithin production. These metabolites have been shown to enhance gut barrier function through multiple mechanisms [76, 97] and reduce inflammatory responses in intestinal cells [76], and may also influence gut microbiome composition [12]. Interestingly, our analysis revealed a positive correlation between the minor metabolite IsoUroA‐S and FC ratios in POMJ‐treated subjects, suggesting that urolithin sulfation patterns may serve as sensitive biomarkers of intestinal microbiome activity. This correlation likely reflects the dynamic interplay between host and microbial metabolism [98, 99], where increased detection of sulfated urolithins may indicate microbiome adaptations to the changing intestinal environment during inflammation resolution [12]. The predominance of metabotype B in our subjects, characterized by production of isourolithin‐A derivatives, aligns with previous studies showing altered urolithin metabolism in dysbiotic conditions [13]. Despite this metabotype profile, POMJ intervention successfully reduced FC levels, highlighting the complex relationship between urolithin metabolism and inflammatory status that extends beyond simple metabotype classification. Systemically, the increase in PBMC inflammatory gene expression might be a compensatory response to maintain immune homeostasis as local inflammation decreases [100]. This dual action could be beneficial in IBD: moderate systemic inflammation might enhance pathogen clearance and tissue repair [101], while reduced local inflammation promotes intestinal mucosal healing. Taken together, these findings suggest a complex interplay between POMJ consumption, ET microbiota metabolism, and both local and systemic inflammatory responses, with urolithins playing a key role. A proposed mechanism for this interaction is illustrated in **Figure** **S7**.

This study has some limitations. First, the low sample size due to patient ineligibility and dropout and the greater heterogeneity that emerged from PBMC transcriptomic analysis in the patients assigned to the active treatment group compared to the control group, even at baseline. Plus, nearly all subjects, although in clinical remission, had ongoing therapy, including treatments with TNF‐α antagonists and other drugs that have been reported to alter immune function [102]. Therefore, given patient heterogeneity in terms of age, disease, and other characteristics, we chose to compare the PMBC transcriptomes at the beginning and end of the intervention for the POMJ and placebo groups independently. This approach, while necessary given the study constraints, means we cannot rule out the possibility that differences in disease status at the beginning of the intervention may have influenced the results. In addition, we observed significant but different response patterns between UC and CD patients in inflammatory markers like FC and endotoxin levels. These differences may reflect the different pathophysiologies of the two conditions—UC is limited to the mucosal layer, whereas CD involves transmural inflammation affecting all gastrointestinal tissue layers [103], potentially resulting in heterogeneous healing patterns [104].

Another limitation is the variability in gut bacterial metabolism of dietary ET‐derived ellagic acid to urolithins, which differs among populations [105, 106]. To address this and evaluate the role of microbiota in postbiotic effects, we identified and quantified ET‐derived metabolites in plasma and urine and performed metabotype profiling. Metabotype B, typically associated with gut dysbiosis [107, 108], characterized most subjects, indicating a stronger impact of IBD‐related factors on the dietary intervention. As ET metabolites are associated with shifts in microbiome composition and functionality [12, 13, 109, 110], additional studies are needed to investigate differences in gut microbiota and fecal urolithin phenotypes in relation to POMJ consumption in patients with IBD.

## 5 Conclusion

Overall, this proof‐of‐principle study demonstrates that 12 weeks of POMJ consumption can significantly modulate the immune and inflammatory response in patients with IBD in clinical remission at high risk of relapse. Although the observed changes in biomarkers and gene expression suggest potential clinical implications, further investigation is needed to translate these findings to definitive clinical outcomes. Long‐term studies are necessary to firmly establish links between the observed molecular changes and sustained remission, reduced hospitalizations and therapy escalation, and improved quality of life for these individuals.

Time‐course analyses across different tissue types to determine if the systemic inflammatory response is transient and how it correlates with clinical outcomes would provide valuable insights into the implications of POMJ consumption in this patient population. These future studies would not only deepen our understanding of POMJ's effects but also help optimize its potential therapeutic application in IBD.

## Conflicts of Interest

The authors declare no conflicts of interest.

## Peer Review

The peer review history for this article is available at https://publons.com/publon/10.1002/mnfr.70067.

## Supporting information



Supporting Information

## Data Availability

Microarray gene expression data discussed in this publication were deposited in the NCBI's Gene Expression Omnibus (GEO) [111] and are accessible through GEO Series accession number GSE242140 (https://www.ncbi.nlm.nih.gov/geo/query/acc.cgi?&acc=%20GSE242140).
