# Supplementary material for: Evidence for a Modulatory Effect of a 12‐Week Pomegranate Juice Intervention on the Transcriptional Response in Inflammatory Bowel Disease Patients Reducing Fecal Calprotectin Levels: Findings From a Proof‐of‐Principle Study
Source: Mol Nutr Food Res. 2025 Apr 21;69(15):e70067. doi: 10.1002/mnfr.70067 (PMC12319513; doi:10.1002/mnfr.70067)
Supplement: Supplementary file 1 — Supporting Information [file MNFR-69-e70067-s001.docx]

**SUPPORTING INFORMATION**

**SUPPLEMENTARY MATERIALS AND METHODS**

**UHPLC Analysis of (Poly)phenolic Compounds in Experimental Beverages**

Pomegranate juice (POMJ) and placebo were extracted and analyzed as previously described [1], with minor modifications. Briefly, thawed samples were centrifuged for 10 min at 14460 × *g* at room temperature (~20−25 °C). Then, the supernatant was diluted (1:20) with methanol:water:formic acid (79:20 :1, v/v), vortexed, filtered using 0.22-μm PVDF filters before UHPLC-ESI-MS^n^ analysis. The samples were analyzed by ultra-high-performance liquid chromatography (UHPLC) coupled with mass spectrometry (MS) using Accela UHPLC 1250 instrument equipped with a linear ion trap MS (LIT-MS) (LTQ XL, Thermo Fisher Scientific Inc., San José, CA, USA) fitted with a heated-ESI (H-ESI-II) probe (Thermo Fisher Scientific Inc.). Separation was carried out by means of an Acquity HSS T3 column (100x 2.1 mm; 1.8 μm particle size; Waters, Milford, MA, USA) installed with a precolumn cartridge, maintained at 40 °C. The mobile phase was pumped at a flow rate of 0.4 mL/min, consisted of a mixture of acidified acetonitrile (0.1% formic acid) (solvent A) and 0.1% aqueous formic acid (solvent B). Following 0.3 min of 5% solvent A in B, the proportion of A was increased linearly to 90% over a period of 6.7 min and maintained for 1.5 min and then the start conditions were re-established in 0.5 min and maintained for 3 min to re-equilibrate the column (total run:12 min). Samples were analyzed in negative ionization mode to identify most of the (poly)phenols, while anthocyanins were identified in positive ionization mode, at the following conditions: capillary temperature of 275 °C, source heater temperature was 200 °C, the sheath gas (N_2_) flow rate was set at 40 (arbitrary units), and the auxiliary gas (N_2_) flow rate at 5, the source, capillary, and tube lens voltages were 4 kV, −42 V, and −118 V, respectively. For anthocyanins, the following conditions were applied: a capillary temperature of 275 °C, source heater temperature of 300 °C, sheath and auxiliary gas (N_2_) flow rates of 40 and 5 units, and source, capillary, and tube lens voltages of 4.5 kV, 20, and 95 V, respectively. Identification was carried out using MS^2^ and MS^3^ information, as previously described by Mena et al. (2012) [1], and as reported in Supplementary Table 1. Quantification was performed using calibration curves constructed using all the available standard compounds. Data processing was performed using XCalibur Version 2.1 (Thermo Fisher Scientific Inc.).

**Validation of Gene Expression Microarray Results by qPCR**

To identify genes for validation of the microarray data by quantitative real-time PCR (qPCR), our candidate genes (*ATF2*, *HSPA14*, *PMS1*, *MTIF3*) were selected. cDNA was obtained using a High-Capacity RNA-to-cDNA Kit (Applied Biosystems, Foster City, CA, USA) following the manufacturer’s instructions. The expression of candidate genes was evaluated using the TaqMan Fast Advanced Master Mix (Applied Biosystems) with TaqMan Gene Expression Assay probes (Applied Biosystems) on a CFX Connect Real-Time PCR Detection System (Bio-Rad Laboratories, Hercules, CA, USA). TaqMan probes are listed in Supplementary Table 3. Samples were run in duplicate using the following program: uracil-N glycosylase incubation at 50 °C for 2 min and polymerase activation at 95 °C for 20 s, followed by 40 cycles (95 °C for 3 s and 60 °C for 30 s). Four reference genes (*GAPDH*, glyceraldehyde-3-phosphate dehydrogenase; *PPIA*, peptidylprolyl isomerase A; *RPLP0*, ribosomal protein lateral stalk subunit P0; *RPS3A*, ribosomal protein S3A) were confirmed as stable genes, with M values < 0.5, and used to normalizing gene expression data by the 2^-ΔΔCq^ method. Data were processed using Bio-Rad CFX Maestro 2.3 Version 5.3.

**SUPPLEMENTARY FIGURES**

**
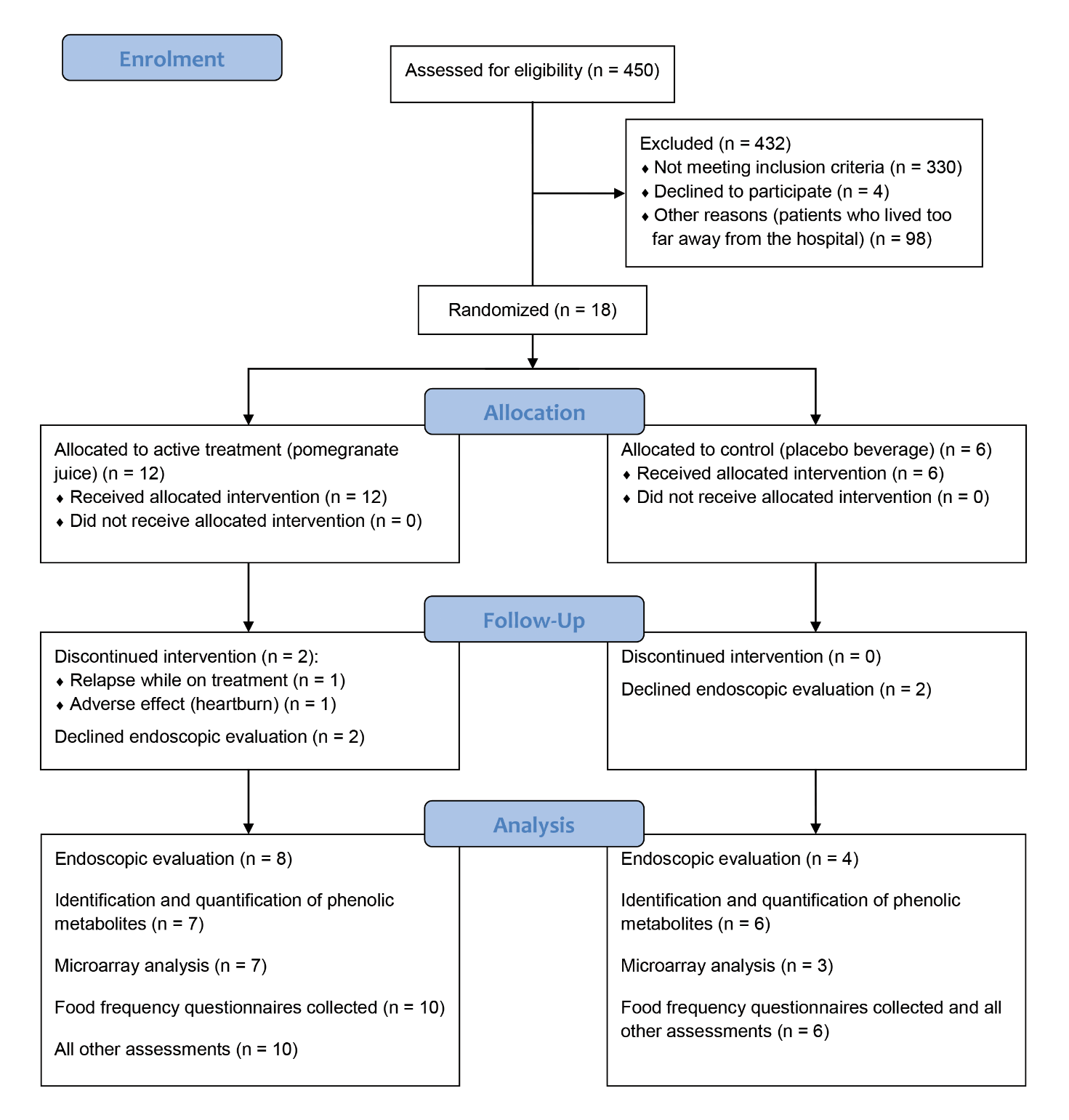
**

**Supplementary Figure 1** CONSORT (Consolidated Standards of Reporting Trials) [48] diagram of participant flow.

**
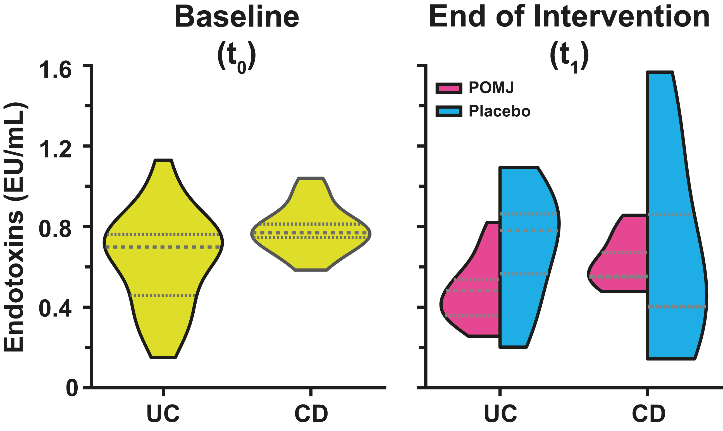
**

**Supplementary Figure 2** Probability density distributions of plasma endotoxin levels in patients with IBD in clinical remission at baseline (t₀) and at the end of the intervention (t₁). The violin plots, generated using kernel density estimation (KDE), illustrate endotoxin distributions in patients with ulcerative colitis (UC) or Crohn's disease (CD) receiving either pomegranate juice (POMJ) or placebo. Horizontal lines within each plot represent the median (dashed) and interquartile range (dotted lines at 25^th^ and 75^th^ percentiles).

**
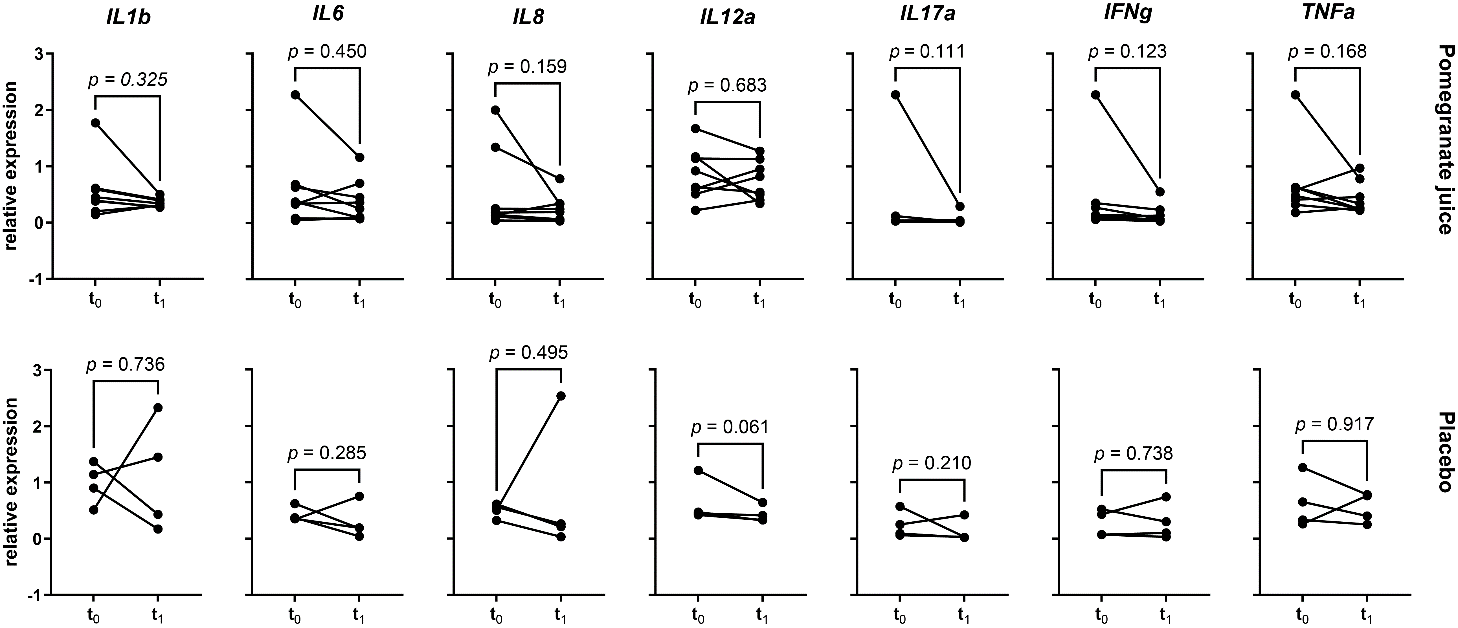
**

**Supplementary Figure 3** Quantitative real-time PCR analysis of the cytokine gene expression in the biopsy of the colon mucosa at baseline (t_0_) and 12 weeks after intervention (t_1_) with pomegranate juice or placebo in patients with IBD in clinical remission. Each line connects a subject’s baseline relative expression (left) to its own endpoint (right). *p*-values were reported from comparisons by ratio-paired t-test. *IFNg*: interferon γ; *IL*: interleukin; *TNFa*: tumor necrosis factor α.


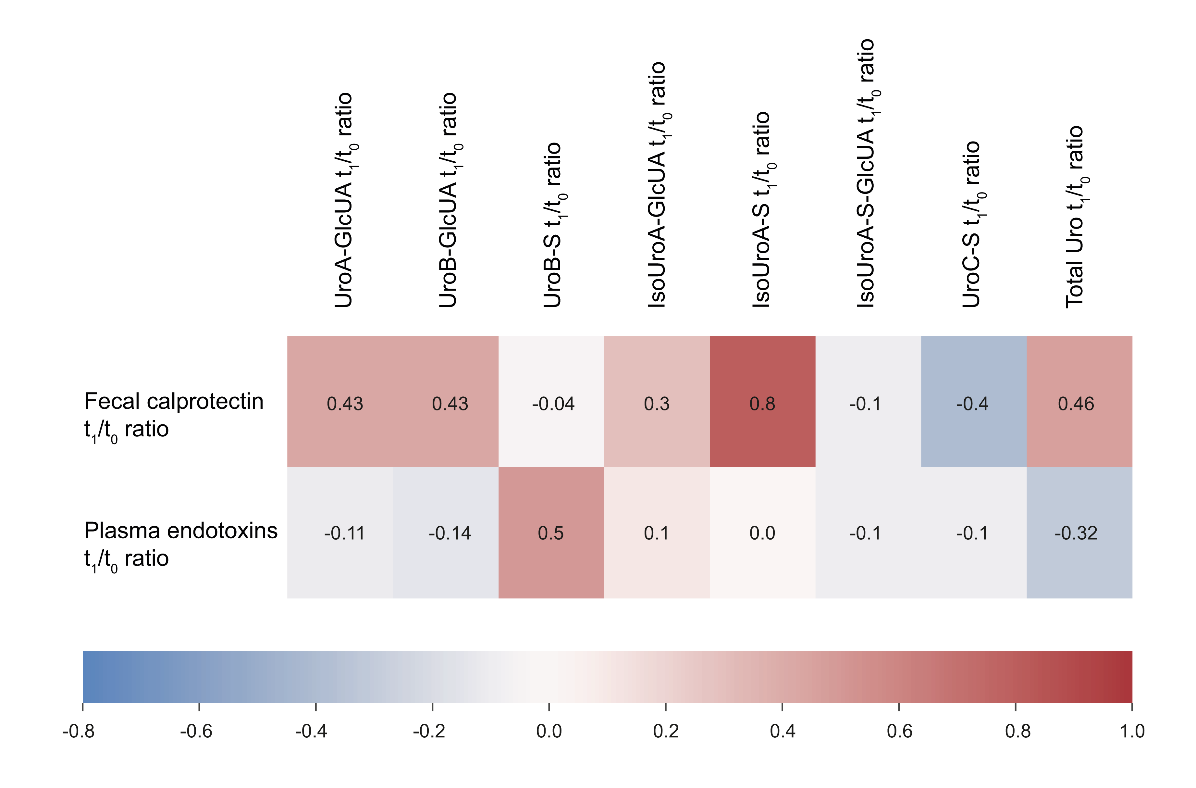


**Supplementary Figure 4** Correlation of changes in urinary urolithin levels with changes in inflammatory markers after 12-week pomegranate juice intervention in patients with IBD in clinical remission. The heatmap shows Spearman correlation coefficients (ρ) between the ratios (t₁/t₀) of urinary urolithins (columns) and the ratios (t₁/t₀) of inflammatory outcomes (rows) that showed significant variation post-intervention (fecal calprotectin and plasma endotoxins). The correlation was considered significant with *p*-values < 0.05 for ρ ≥ 0.64 or ≤ −0.64 [2]. IsoUroA-GlcUA: isourolithin A- glucuronide; IsoUroA-S: isourolithin A-sulfate; IsoUroA-S-GlcUA: Isourolithin A-sulfate-glucuronide; Total Uro: total urolithins; UroA-GlcUA: urolithin A-glucuronide; UroB-GlcUA: urolithin B-glucuronide; UroB-S: urolithin B-sulfate; UroC-S: urolithin C-sulfate.

**Supplementary Figure 5** Normalized expression values of genes differentially expressed in peripheral blood mononuclear cells of patients in the placebo group and involved in canonical pathways with z-score > 0 and *p*-value ≤ 0.01, as determined by Ingenuity Pathway Analysis and indicated by row annotation. Rows are centered; unit variance scaling is applied to rows. Rows are clustered using Euclidean distance and complete linkage. cAMP: cyclic adenosine monophosphate; eNOS: endothelial nitric oxide synthase; HIF1a: hypoxia inducible factor 1 subunit α; ILK: integrin-linked kinase; NO: nitric oxide; PKA: protein kinase A; POMJ: pomegranate juice.

**Supplementary Figure** **6** Normalized expression values of genes differentially expressed in peripheral blood mononuclear cells of patients in the pomegranate juice (POMJ) group and located upstream to (left) “inflammatory response” or (right) “Inflammation of gastrointestinal tract”. The predicted downstream effect of each gene on the function, according to Ingenuity Pathway Analysis, is reported. Rows are centered; unit variance scaling is applied to rows. Rows are clustered using Euclidean distance and complete linkage.


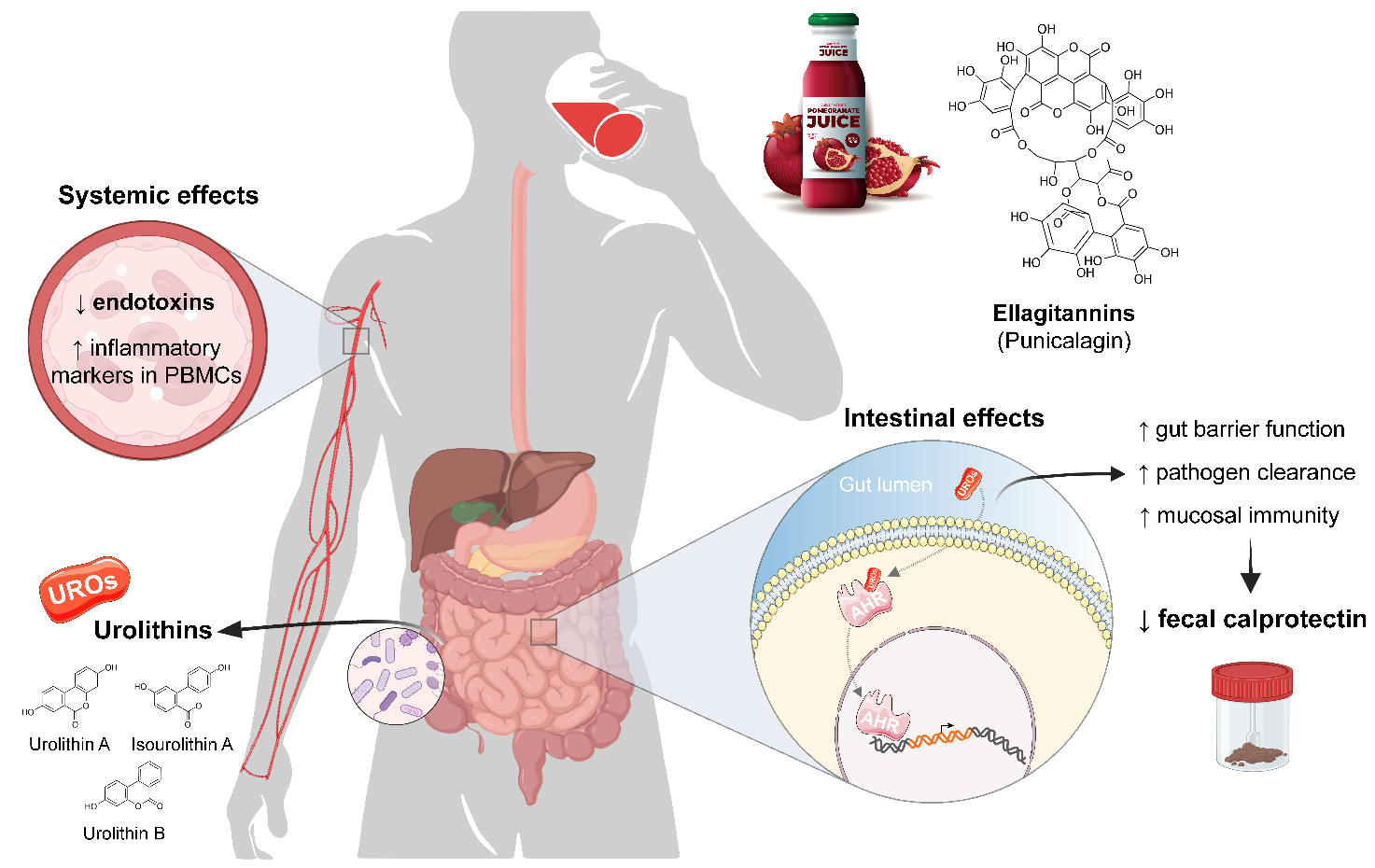


**Supplementary Figure 7** Proposed mechanisms of action for pomegranate juice (POMJ) in patients with IBD in clinical remission, integrating observed effects, Ingenuity Pathway Analysis (IPA) predictions and literature knowledge [3]. Gut microbiota metabolizes ellagitannins (punicalagin) from POMJ to ellagic acid and urolithins (UROs), which exert both local intestinal and systemic effects. In the gut, urolithins modulate the aryl hydrocarbon receptor (AHR) [3]; IPA predicts improved barrier function, enhanced mucosal immunity, and improved pathogen clearance, which together contribute to reduced fecal calprotectin levels. Improved barrier function leads to reduced plasma endotoxin levels. In addition, an increase in systemic inflammatory response is predicted, potentially representing a compensatory immune response. This dual action of localized anti-inflammatory effects in the gut and modulated systemic inflammatory response may contribute to the maintenance of remission in patients with quiescent IBD. Figure partially created with BioRender.com, incorporating original images.

**SUPPLEMENTARY TABLES**

**Supplementary Table 1** Chromatographic and spectrometric properties of the monitored metabolites. ‘STD’ means that the reference standard is available for quantification. Fragments were ordered by abundance, and the most abundant MS^2^ was chosen for MS^3^ fragmentation.

| **ID Compounds** | **RT (min)** | **[M−H]^−^**  **(*m/z*)** | **MS^2^ ion fragments (*m/z*) ^c^** | **MS^3^ ion fragments (*m/z*) ^c^** |
| --- | --- | --- | --- | --- |
| Punicalagin-like | 0.67 | 1101 | 1083, 301, 781, 601 | 781, 601, 575, 721 |
| HHDP-hexoside | 1.03 | 481 | 301, 275, 257, 363, 391 | 257, 229, 273,185 |
| Galloyl-hexoside (STD) | 1.07 | 331 | 169, 241, 125 | - |
| Galloyl-HHDP-gluconate (lagerstannin C) | 1.17 | 649 | 301, 497 | 257, 229 |
| Gallic acid (STD) | 1.32 | 169 | 125 | - |
| Punicalin α/A | 1.51 | 781 | 601, 721, 575, 677 | 299, 271, 215, 243 |
| Punicalin β/B | 1.55 | 781 | 601, 721, 299, 449 | 299, 271, 583, 243, 215 |
| Galloyl-HHDP-gluconate | 1.95 | 649 | 497, 301 | - |
| Punicalagin isomer | 2.17 | 1083 | 1065, 807, 601, 721 | 721, 575, 1047, 1021, 601 |
| Delphinidin 3,5-*O*-diglucoside | 2.20 | [M]^+^: 627 | 465, 303 | - |
| Cyanidin 3,5-*O*-diglucoside | 2.36 | [M]^+^: 611 | 449, 287 | - |
| Hydroxybenzoic acid-hexoside | 2.43 | 299 | 239, 179, 137, 209, 93 | 179, 137, 93 |
| Datiscetin-hexoside | 2.44 | 447 | 285, 267 | 241, 257, 125, 217, 243 |
| Punicalagin α (STD) | 2.58 | 1083 | 781, 601 | 601, 721 |
| Pelargonidin 3,5-*O*-diglucoside | 2.58 | [M]^+^: 595 | 433, 271 | - |
| Delphinidin 3-*O*-glucoside (STD) | 2.62 | [M]^+^: 465 | 303 | - |
| Pedunculagin I isomer | 2.67 | 783 | 481, 721, 765, 301 | - |
| Cyanidin 3-*O*-glucoside (STD) | 2.74 | [M]^+^: 449 | 287 | - |
| Punicalagin β (STD) | 2.75 | 1083 | 781, 601 | 601, 721 |
| Kaempferol-hexoside | 2.80 | 447 | 285, 284, 327, 255 | 257, 267, 229, 241 |
| Pelargonidin 3-*O*-glucoside | 2.90 | [M]^+^: 433 | 271 | - |
| Galloyl-HHDP-hexoside | 3.03 | 633 | 301, 463, 275, 481 | 301, 257, 229 |
| Ellagic acid-hexoside | 3.15 | 463 | 301 | 301, 257, 229 |
| Dihydrokaempferol-hexoside | 3.18 | 449 | 287, 269, 259,431 | 259, 243, 269, 201 |
| Vanillic acid (STD) | 3.30 | 167 | 125, 123, 151 | 123, 108, 81, 95 |
| Quercetin-rutinoside (rutin) (STD) | 3.31 | 463 | 301 | 179, 151, 257, 301, 273 |
| Syringetin-hexoside | 3.31 | 507 | 327, 345, 315 | 312, 283 |
| Ellagic acid-pentoside | 3.32 | 433 | 301 | 300, 257, 229 |
| Ellagic acid-deoxyhexoside | 3.41 | 447 | 300, 301, 285, 373 | 257, 229, 243, 216 |
| Coumaric acid derivative | 3.46 | 429 | 163, 265,325 | 145, 119, 103, 89 |
| Ellagic acid (STD) | 3.58 | 301 | 301, 257, 229, 185 | 229, 185 |
| Secoisolariciresinol-hexoside | 3.65 | 523 | 361, 347 | 346, 165, 179, 313 |
| Phloretin-hexoside (phlorizin) (STD) | 4.16 | 435 | 273, 297 | 167 |
| Pinocembrin | 4.65 | 255 | 213, 211, 151, 145, 227, 187 | 213, 211, 151, 187, 169, 141, 145, 195 |

CE: collision energy; HHDP: hexahydroxydiphenoyl; RT: retention time.

**Supplementary Table 2** The minimum detectable dose (MDD) of each analyte for the Quantikine ELISA Immunoassay (CRP) and the Magnetic Luminex Performance Assay (IL-1β, IL-6, IL-8, IL-10, and TNF-α) (R&D Systems Inc., Minneapolis, MN, USA).

| **Analyte** | **MDD range** | **Mean MDD** |
| --- | --- | --- |
| CRP | 0.005–0.022 ng/mL | 0.010 ng/mL |
| IL-1β | 0.03–0.18 pg/mL | 0.08 pg/mL |
| IL-6 | 0.08–0.31 pg/mL | 0.14 pg/mL |
| IL-8 | 0.02–0.07 pg/mL | 0.04 pg/mL |
| IL-10 | 0.066–0.671 pg/mL | 0.211 pg/mL |
| TNF-α | 0.13–0.54 pg/mL | 0.29 pg/mL |

CRP: C-reactive protein; IL: interleukin; TNF-α: tumor necrosis factor α.

**Supplementary Table 3** TaqMan assays used for gene expression analysis (Applied Biosystems, Foster City, CA, USA).

| **Gene symbol** | **Gene name** | **TaqMan Assay ID** |
| --- | --- | --- |
| **Target genes** |  |  |
| *ATF2* | activating transcription factor 2 | Hs01095345_m1 |
| *HSPA14* | heat shock protein family A (Hsp70) member 14 | Hs01102298_g1 |
| *IFN-γ* | interferon gamma | Hs00989291_m1 |
| *IL-1β* | interleukin 1 beta | Hs01555410_m1 |
| *IL-6* | interleukin 6 | Hs00174131_m1 |
| *IL-8* | interleukin 8 | Hs00174103_m1 |
| *IL-12a* | interleukin 12A | Hs01073447_m1 |
| *IL-17a* | interleukin 17A | Hs00174383_m1 |
| *MTIF3* | mitochondrial translational initiation factor 3 | Hs00794538_m1 |
| *PMS1* | PMS1 homolog 1 | Hs00922262_m1 |
| *TNF-α* | tumor necrosis factor alpha | Hs00174128_m1 |
| **Reference genes** |  |  |
| *B2M* | beta-2-microglobulin | Hs99999907_m1 |
| *GAPDH* | glyceraldehyde-3-phosphate dehydrogenase | Hs99999905_m1 |
| *HPRT* | hypoxanthine guanine phosphoribosyl transferase | Hs99999909_m1 |
| *PPIA* | peptidylprolyl isomerase A | Hs99999904_m1 |
| *RPLP0* | ribosomal protein lateral stalk subunit P0 | Hs99999902_m1 |
| *RPS3A* | ribosomal protein S3A | Hs00832893_sH |

**Supplementary Table 4** Chromatographic and spectrometric properties of the monitored metabolites.

| **Compound** | **RT (min)** | **Parent ion [M-H]¯ *(m/z)*** | **S-Lens** | **Quantifier** | | **Qualifier(s)** | | **Standard used for quantification** |
| --- | --- | --- | --- | --- | --- | --- | --- | --- |
|  |  |  |  | **Product ion (*m/z*)** | **CE (V)** | **Product ion (*m/z*)** | **CE (V)** |  |
| Isourolithin A (IsoUroA) | 5.5 | 227 | 92 | 198 | 36 | 171, 181, 183, 159 | 29, 30, 24, 28 | Not found |
| Urolithin B (UroB) | 6.22 | 211 | 116 | 167 | 27 | 139, 117 | 34, 36 | Not found |
| Urolithin C (UroC) | 5.05 | 243 | 122 | 187 | 32 | 171, 199 | 29, 29 | Not found |
| Urolithin D (UroD) | – | 259 | 109 | 242 | 29 | 213, 241 | 33, 25 | Not found |
| Dimethylellagic acid-glucuronide (DMEAG) | 5.15 | 505 | 78 | 329 | 17 | 314, 113 | 35, 20 | IsoUroA-3-GlcUA |
| Isourolithin A-sulfate (IsoUroA-S) | 6.68 | 307 | 78 | 227 | 19 | – | – | UroA-8-S |
| Urolithin A-glucuronide (UroA-GlcUA) ^†^ | 4.89 | 403 | 78 | 227 | 37 | 113 | 15 | UroA-8-GlcUA |
| Isourolithin A-glucuronide (IsoUroA-GlcUA) ^†^ | 4.97 | 403 | 78 | 227 | – | 113 | 15 | IsoUroA-3-GlcUA |
| Isourolithin A-sulfate-glucuronide (IsoUroA-S-GlcUA) | 6.9 | 483 | 78 | 307 | 37 | 227, 403 | 15, 19 | IsoUroA-3-GlcUA |
| Urolithin B-glucuronide (UroB-GlcUA) ^†^ | 5.38 | 387 | 83 | 211 | 35 | 113 | 17 | UroB-GlcUA |
| Urolithin B-sulfate (UroB-S) | 7.5 | 291 | 78 | 211 | 19 | – | – | UroA-8-S |
| Urolithin C-glucuronide (UroC-GlcUA) | 4.9 | 419 | 78 | 243 | 37 | 113 | 15 | IsoUroA-3-GlcUA |
| Urolithin C-sulfate (UroC-S) | 6.45 | 323 | 78 | 243 | 19 | – | – | UroA-8-S |
| Urolithin D-glucuronide (UroD-GlcUA) | – | 435 | 78 | 259 | 37 | 113 | 37 | Not found |
| Urolithin D-sulfate (UroD-S) | – | 339 | 78 | 259 | 19 | – | – | Not found |

^†^Compounds used for metabotyping. Abbreviations for the compound names are reported in parentheses. CE: collision energy; RT: retention time.

**Supplementary Table 5** Nutrient composition of the experimental beverages, per 100 mL. The beverages were supplied by Conserve Italia (Bologna, Italy).

| **Nutrient** | **Pomegranate juice** | **Placebo** |
| --- | --- | --- |
| Water (g) | 84.09 | 83.65 |
| Energy (kcal) | 63 | 67 |
| Protein (g) | 0.26 | 0.10 |
| Fat (g) | 0.17 | 0.26 |
| Carbohydrates (g) | 15.05 | 16.09 |
| Sugars, total (g) | 13.42 | 15.41 |
| Fiber, total (g) | < 0.50 | < 0.50 |
| Ash (g) | 0.43 | 0.10 |
| Vitamin C (ascorbic acid) (mg) | < 1.00 | < 1.00 |
| Citric acid (g) | 0.99 | 1.86 |

**Supplementary Table 6** (Poly)phenolic composition of the pomegranate juice, per 100 mL (mean ± SD, n = 3).

| **Compound** | **Concentration (mg/100 mL)** |
| --- | --- |
| **Ellagitannins** |  |
| Punicalagin-α | 5.62 ± 0.73 |
| Punicalagin-β | 10.90 ± 0.71 |
| Punicalagin isomer | 1.19 ± 0.68 |
| Punicalagin-like | 9.49 ± 0.70 |
| Punicalin-α | 11.73 ± 6.25 |
| Punicalin-β | 8.91 ± 3.09 |
| HHDP-hexoside | 14.59 ± 0.99 |
| Galloyl-hexoside | 13.07 ± 0.61 |
| Galloyl-HHDP-gluconate (lagerstanin-C) | 4.32 ± 1.44 |
| Galloyl-HHDP-gluconate | 2.27 ± 1.42 |
| Pedunculagin I isomer | 3.86 ± 1.07 |
| Galloyl-HHDP-hexoside | 21.28 ± 1.60 |
| Ellagic acid | 58.19 ± 7.71 |
| Ellagic acid-hexoside | 99.68 ± 4.71 |
| Ellagic acid-pentoside | 2.32 ± 0.30 |
| Ellagic acid-deoxyhexoside | 23.89 ± 1.05 |
| *Total ellagitannins* | *291.29 ± 17.50* |
| **Anthocyanins** |  |
| Cyanidin-3-*O*-glucoside | 10.96 ± 0.14 |
| Cyanidin-3,5-*O*-diglucoside | 15.20 ± 0.37 |
| Pelargonidin-3-*O*-glucoside | 2.02 ± 0.30 |
| Pelargonidin-3,5-*O*-diglucoside | 1.87 ± 0.28 |
| Delphinidin-3-*O*-glucoside | 0.37 ± 0.02 |
| Delphinidin-3,5-*O*-diglucoside | 0.72 ± 0.11 |
| *Total anthocyanins* | *31.14 ± 1.23* |
| **Chalcone** |  |
| Phloretin-hexoside (phlorizin) | 1.91 ± 0.22 |
| **Flavanone** |  |
| Pinocembrin | 0.04 ± 0.00 |
| **Flavone** |  |
| Datiscetin-hexoside | 1.64 ± 0.68 |
| **Flavonol** |  |
| Kaempferol-hexoside | 0.39 ± 0.10 |
| Dihydrokaempferol-hexoside | 0.98 ± 0.05 |
| Quercetin-rutinoside (rutin) | 0.08 ± 0.09 |
| Syringetin-hexoside | 3.12 ± 0.43 |
| *Total flavonols* | *4.57 ± 0.41* |
| **Hydroxybenzoic acid** |  |
| Vanillic acid | 3.29 ± 0.42 |
| Gallic acid | 1.75 ± 0.33 |
| Hydroxybenzoic acid-hexoside | 1.79 ± 0.24 |
| *Total hydroxybenzoic acid* | *6.82 ± 0.59* |
| **Hydroxycinnamic acid** |  |
| Coumaric acid derivate | 20.20 ± 1.86 |
| **Lignan** |  |
| Secoisolariciresinol-hexoside | 0.13 ± 0.01 |
| *Total (poly)phenols* | *359.74 ± 18.40* |

HHDP: hexahydroxydiphenoyl.

**Supplementary Table 7** Disease activity scores at baseline (t_0_) and 12 weeks after intervention (t_1_) with pomegranate juice (POMJ) or placebo in patients with IBD in clinical remission. *p*-values were reported from comparisons using the ratio-paired t-test.

| **Disease activity scores** | Median (IQR)  POMJ t_0_ | Median (IQR)  POMJ t_1_ | *p*-value | Median (IQR)  Placebo t_0_ | Median (IQR)  Placebo t_1_ | *p*-value |
| --- | --- | --- | --- | --- | --- | --- |
| Mayo Endoscopic Subscore (MES) | 1.00 (0.00–1.00) | 1.00 (1.00–1.00) | 0.3739 | 1.00 (1.00–1.00) | 1.00 (0.75–1.00) | > 0.9999 |
| Simple Clinical Colitis Activity Index (SCCAI) ^†^ | 1.00 (1.00–3.50) | 1.00 (0.00–1.50) | 0.2317 | 1.00 (0.50–1.50) | 0.00 (0.00–1.00) | > 0.9999 |
| Crohn’s Disease Activity Index (CDAI) ^‡^ | 36.00 (33.30–48.50) | 37.80 (31.50–39.40) | 0.4544 | 52.60 (44.70–59.60) | 30.80 (23.65–53.30) | 0.2832 |

^†^ only for patients with UC; ^‡^ only for patients with CD. Zero values were excluded from statistical analysis. IQR: interquartile range (Q1 to Q3).

**Supplementary Table 8** Plasma levels of trimethylamine *N*-oxide (TMAO) and its precursors at baseline (t_0_) and 12 weeks after intervention (t_1_) with pomegranate juice (POMJ) or placebo in patients with IBD in clinical remission. *p*-values are reported from comparisons by ratio-paired t-test; significant values (< 0.05) are marked in bold.

| **Plasma TMAO and TMAO precursors (μM)** | Median (IQR)  POMJ t_0_ | Median (IQR)  POMJ t_1_ | *p*-value | Median (IQR)  Placebo t_0_ | Median (IQR)  Placebo t_1_ | *p*-value |
| --- | --- | --- | --- | --- | --- | --- |
| TMAO | 4.08 (2.52–4.80) | 3.71 (3.39–4.28) | 0.9923 | 4.13 (2.80–4.63) | 4.30 (3.03–10.00) | 0.2058 |
| Carnitine | 49.54 (47.33–54.60) | 49.67 (46.77–55.61) | 0.9896 | 47.47 (41.14–54.36) | 52.86 (43.26–57.86) | 0.9284 |
| Dimethylglycine | 4.07 (3.49–4.46) | 4.48 (3.20–5.16) | 0.0926 | 4.33 (3.20–4.95) | 4.54 (3.37–5.46) | 0.3052 |
| Betaine | 23.74 (18.56–25.19) | 20.82 (19.18–25.78) | 0.4874 | 23.33 (20.52–27.68) | 25.39 (21.93–29.42) | **0.0388** |
| Choline | 23.24 (20.62–29.37) | 22.68 (21.54–26.84) | 0.4875 | 29.42 (22.54–35.69) | 26.54 (23.06–28.55) | 0.9005 |

IQR: interquartile range (Q1 to Q3); TMAO: trimethylamine *N*-oxide.

**Supplementary Table 9** Plasma metabolites were characterized and quantified at baseline (t_0_) and 12 weeks after intervention (t_1_) with pomegranate juice (POMJ) or placebo in patients with IBD in clinical remission. *p*-values were reported from comparisons using the ratio-paired t-test.

| **Urolithin (μM)** | Median (IQR)  POMJ t_0_ ^†^ | Median (IQR)  POMJ t_1_ ^†^ | *p*-value | Median (IQR)  Placebo t_0_ | Median (IQR)  Placebo t_1_ | *p*-value |
| --- | --- | --- | --- | --- | --- | --- |
| UroB-GlcUA | 0.000 (0.000–0.008) | 0.015 (0.000–0.071) | 0.9656 | 0.000 (0.000–0.000) | 0.000 (0.000–0.000) | > 0.9999 |
| UroB-S | 0.000 (0.000–0.000) | 0.000 (0.000–0.000) | > 0.9999 | 0.000 (0.000–0.000) | 0.000 (0.000–0.000) | > 0.9999 |
| UroA-GlcUA | 0.000 (0.000–0.023) | 0.024 (0.001–0.109) | 0.7183 | 0.000 (0.000–0.000) | 0.000 (0.000–0.000) | > 0.9999 |
| IsoUroA-GlcUA | 0.000 (0.000–0.008) | 0.023 (0.000–0.037) | 0.7119 | 0.000 (0.000–0.000) | 0.000 (0.000–0.000) | > 0.9999 |
| IsoUroA-S | 0.000 (0.000–0.000) | 0.000 (0.000–0.000) | > 0.9999 | 0.000 (0.000–0.000) | 0.000 (0.000–0.000) | > 0.9999 |
| IsoUroA-S-GlcUA | 0.000 (0.000–0.000) | 0.000 (0.000–0.000) | > 0.9999 | 0.000 (0.000–0.000) | 0.000 (0.000–0.000) | > 0.9999 |
| UroC-GlcUA | 0.000 (0.000–0.000) | 0.000 (0.000–0.000) | > 0.9999 | 0.000 (0.000–0.001) | 0.000 (0.000–0.000) | > 0.9999 |
| UroC-S | n.d. | n.d. | – | n.d. | n.d. | – |
| DMEAG | 0.000 (0.000–0.002) | 0.002 (0.000–0.009) | 0.8559 | 0.000 (0.000–0.000) | 0.000 (0.000–0.000) | > 0.9999 |
| *Total urolithins* | *0.002 (0.000–0.039)* | *0.129 (0.002–0.192)* | *0.7832* | *0.000 (0.000–0.000)* | *0.000 (0.000–0.000)* | *> 0.9999* |

^†^ Three subjects were excluded from the analysis because plasma samples were not available for both t_0_ and t_1_. Zero values were excluded from statistical analysis. DMEAG: dimethylellagic acid-glucuronide; IQR: interquartile range (Q1 to Q3); IsoUroA-GlcUA: isourolithin A- glucuronide; IsoUroA-S: isourolithin A-sulfate; IsoUroA-S-GlcUA: isourolithin A-sulfate-glucuronide; n.d.: non detected; UroA-GlcUA: urolithin A-glucuronide; UroB-GlcUA: urolithin B-glucuronide; UroB-S: urolithin B-sulfate; UroC-GlcUA: urolithin C-glucuronide; UroC-S: urolithin C-sulfate.

**Supplementary Table 10** Urolithin metabotypes at baseline (t_0_) and 12 weeks after intervention (t_1_) with pomegranate juice (POMJ) or placebo in patients with IBD in clinical remission.

| **Urolithin metabotypes** | POMJ t_0_ ^†^ | POMJ t_1_ ^†^ | Placebo t_0_ | Placebo t_1_ |
| --- | --- | --- | --- | --- |
| UM-0 | – | – | 1 (17%) | – |
| UM-A | 1 (14%) | – | – | – |
| UM-B | 6 (86%) | 7 (100%) | 5 (83%) | 6 (100%) |

^†^ Three subjects were excluded from the analysis because samples were not available for both t_0_ and t_1_. UM-0: urolithin metabotype 0; UM-A: urolithin metabotype A; UM-B: urolithin metabotype B.

**Supplementary Table 11** Changes in gene expression in peripheral blood mononuclear cells of the study subjects were observed using microarray-based transcriptomic analysis and quantitative real-time PCR. *p*-values were reported from comparisons by paired t-test; significant fold changes and *p*-values (log_2_ fold-change ≥ 0.5 or ≤ −0.5; *p-*values < 0.05) are marked in bold.

|  | POMJ t_1_ vs t_0_ | | | | placebo t_1_ vs t_0_ | | | |
| --- | --- | --- | --- | --- | --- | --- | --- | --- |
|  | microarray analysis | | qPCR analysis | | microarray analysis | | qPCR analysis | |
| **Gene** | log_2_ fold-change | *p*-value | log_2_ fold-change | *p*-value | log_2_ fold-change | *p*-value | log_2_ fold-change | *p*-value |
| *ATF2* | −0.430 | **0.001** | −0.437 | **0.045** | 0.011 | 0.861 | −0.111 | 0.435 |
| *HSPA14* | −0.312 | **0.001** | −0.425 | **0.030** | −0.028 | 0.680 | −0.313 | **0.042** |
| *MTIF3* | −0.203 | **0.008** | −0.401 | **0.042** | −0.047 | 0.753 | −0.009 | 0.940 |
| *PMS1* | −0.614 | **0.002** | −0.536 | **0.018** | −0.145 | 0.575 | −0.129 | 0.165 |

ATF2: activating transcription factor 2; HSPA14: heat shock protein family A (Hsp70) member 14; MTIF3: mitochondrial translational initiation factor 3; PMS1: PMS1 homolog 1; POMJ: pomegranate juice; qPCR: quantitative real-time PCR.

**REFERENCES**

[1] P. Mena, L. Calani, C. Dall'Asta, G. Galaverna, C. García-Viguera, R. Bruni, A. Crozier, D. Del Rio, *Molecules* **2012**, *17*, 14821.

[2] D. G. Bonett, T. A. Wright, *Psychometrika* **2000**, *65*, 23.

[3] C. J. G. Pinto, M. A. Ávila-Gálvez, Y. Lian, P. Moura-Alves, C. Nunes Dos Santos, *Redox Biol.* **2023**, *61*, 102622.
